# Methotrexate–Cyclodextrin Systems: Molecular Recognition, Formulation Design, and Translational Perspectives

**DOI:** 10.3390/cimb48090905

**Published:** 2026-09-04

**Authors:** Konrad Adam Michalik, Dominik Grzywacz, Łukasz Szeleszczuk

**Affiliations:** 1 Department of Organic and Physical Chemistry, Faculty of Pharmacy, Medical University of Warsaw, 02-097 Warsaw, Poland; konrad.michalik@student.wum.edu.pl; 2 Faculty of Chemistry, University of Warsaw, 02-093 Warszawa, Poland; d.grzywacz4@student.uw.edu.pl

**Keywords:** methotrexate, cyclodextrin, inclusion complex, β-cyclodextrin, host-guest chemistry, drug delivery, hydrogel, rheumatoid arthritis

## Abstract

Methotrexate (MTX) remains central to the treatment of rheumatoid arthritis and several malignancies, yet its use is complicated by dose-dependent toxicity, variable oral exposure, photolability, and pH-dependent solubility. Cyclodextrins (CDs) can alter the molecular environment of MTX, but the literature often conflates true inclusion complexes with formulations in which CD merely forms part of a larger carrier. This review critically distinguishes direct MTX–CD complexes, dosage forms built from a preformed complex, CD-containing carriers without direct evidence of cavity occupancy, and covalent MTX–CD conjugates. Particular attention is given to binding stoichiometry, apparent association constants, guest orientation, preparation methods, and the evidence needed to establish inclusion. Both solution-state host–guest association and isolated solid products are considered; however, solid-state changes are treated as supportive evidence rather than as stand-alone proof of cyclodextrin cavity occupancy. Among the limited head-to-head comparisons of native cyclodextrins, β-CD generally showed more favorable MTX recognition than α- or γ-CD, although the magnitude of this difference is method- and condition-dependent. Complexation can improve dissolution, photostability, and oral or local delivery; however, greater solubilization does not necessarily enhance membrane transport. In carrageenan hydrogels, β-CD increased MTX loading and release while reducing membrane permeation, illustrating the importance of the equilibrium between complexed and freely permeating drug. The most promising systems remain preclinical. Progress toward translation will require clearer nomenclature, orthogonal structural characterization, mechanism-resolving controls, and standardized pharmacokinetic and safety studies.

## 1. Introduction

Methotrexate (MTX) occupies an unusual position in therapeutics. At low weekly doses it remains the anchor conventional synthetic disease-modifying antirheumatic drug for rheumatoid arthritis, while at substantially higher exposures it is used for hematologic malignancies, osteosarcoma, trophoblastic disease, and selected central nervous system tumors. The 2025 EULAR update and the most recent ACR pharmacologic treatment guideline, published in 2021, continue to place MTX near the center of initial treatment strategies, although escalation is guided by disease activity, comorbidity, tolerance, and access to targeted therapies [1,2]. Its pharmacology is equally context-dependent. Antiproliferative activity is linked to folate antagonism and inhibition of nucleotide synthesis, whereas anti-inflammatory effects also involve intracellular polyglutamation, inhibition of purine metabolism, and increased extracellular adenosine signaling [3,4,5].

The therapeutic breadth of MTX is matched by a narrow margin for error in several clinical settings. High-dose regimens can be complicated by delayed renal clearance, mucositis, marrow suppression, hepatic injury, and prolonged systemic exposure. Prevention and management of high-dose MTX toxicity therefore depend on adequate hydration and urinary alkalinization, serial monitoring of plasma MTX concentrations and renal function, pharmacokinetically guided leucovorin rescue, and timely use of glucarpidase when delayed MTX elimination with clinically relevant overexposure or renal dysfunction occurs [6,7]. In long-term inflammatory disease, the problems are different but no less relevant: gastrointestinal intolerance, inconsistent absorption, hepatic monitoring, and treatment of fatigue can erode adherence. Formulation strategies are therefore attractive when they increase useful exposure at the site of action without simply raising the circulating concentration of free drug.

Cyclodextrins are established pharmaceutical excipients with a characteristic truncated cone architecture. The common native forms contain six, seven, or eight α-(1,4)-linked glucopyranose units and are designated α-, β-, and γ-CD, respectively. Their hydrophilic exterior and less polar internal cavity permit suitably shaped molecular fragments to form reversible, non-covalent host–guest associations. The behavior of these complexes depends on cavity size, guest ionization, cyclodextrin substitution, solvent composition, and competition from other formulation components [8,9,10].

In pharmaceutical systems, CD association can influence apparent solubility, dissolution, chemical and photochemical stability, drug release, and the organization of supramolecular or nanoparticulate carriers [9,10]. These effects are not uniformly beneficial. Increasing total dissolved drug can reduce the thermodynamic activity of the freely permeating species when complex dissociation is insufficiently rapid, while CD aggregates and nonideal phase solubility behavior can further complicate concentrated formulations [11,12,13].

Research on MTX and CDs has expanded from early chromatographic comparisons and a simple β-CD inclusion complex to alkylated and carbohydrate-substituted hosts, dimers, polymers, ternary systems, niosomes, hydrogels, nanosponges, metal–organic frameworks, magnetic particles, and stimulus-responsive assemblies. As the materials became more elaborate, the terminology became less precise. In some reports, “MTX–CD complex” denotes a characterized host–guest pair. In others, CD forms the carrier, complexes a different component such as cholesterol or azobenzene, or serves as a surface ligand, while the location of MTX remains uncertain.

**Figure 1:**
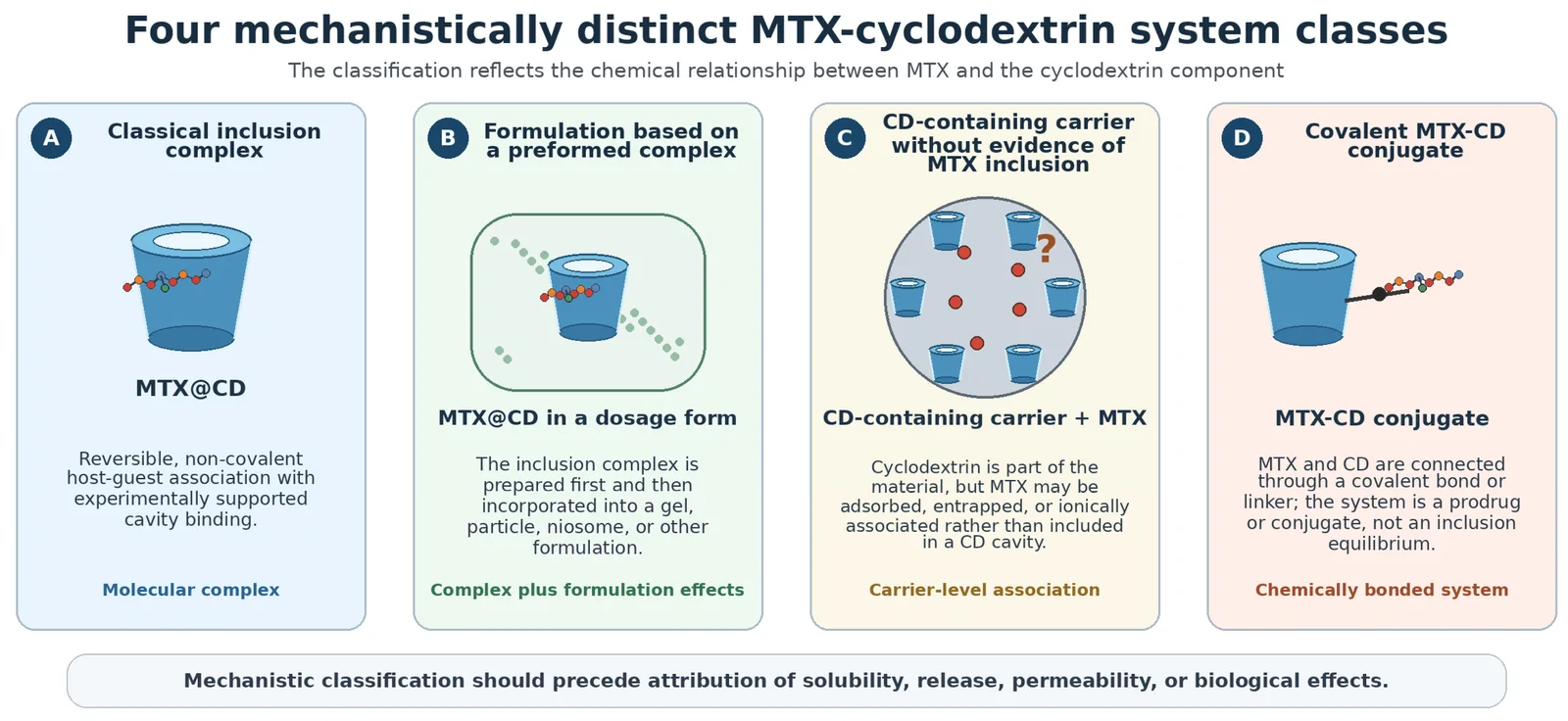
Mechanistic classification of MTX–cyclodextrin systems used in this review. (**A**) Direct reversible MTX–CD inclusion complex: MTX is non-covalently associated with a chemically defined cyclodextrin host, with experimental evidence supporting molecular binding and, where available, cavity occupancy. (**B**) Formulation based on a preformed MTX–CD complex: the molecular complex is prepared and characterized before incorporation into a secondary dosage form or carrier; its unchanged persistence in the final formulation is not assumed. (**C**) CD-containing carrier without demonstrated MTX inclusion: cyclodextrin is a structural or functional component of the carrier, whereas MTX may be loaded, adsorbed, encapsulated, or partitioned into another domain without direct evidence of CD cavity occupancy. (**D**) Covalent MTX–CD conjugate: MTX is chemically linked to the cyclodextrin scaffold and is therefore treated as a conjugate or prodrug rather than as a reversible inclusion complex. The schematic illustrates mechanistic relationships and is not drawn to scale. Original figure created for this review with limited AI-assisted (Chat GPT, OpenAI, version 5.6) graphical drafting; all scientific content and category definitions were developed and verified by the authors.

To keep chemical mechanisms separate from formulation performance, this review uses four categories. A direct MTX–CD complex is a reversible molecular association supported by evidence that MTX binds to the CD host. A formulation based on a preformed complex contains such a complex within a second delivery platform. A CD-containing carrier uses CD as a structural or functional component but lacks direct proof that MTX occupies the cavity. A covalent MTX–CD conjugate contains a chemical bond between the drug and the carbohydrate scaffold and should be discussed as a prodrug or conjugate rather than as an inclusion complex (Figure 1).

Against this background, the present review examines how cyclodextrin structure, complex preparation route, and formulation architecture shape the behavior of MTX. Particular attention is paid to the strength of the evidence for cavity inclusion, the distinction between molecular and carrier-level effects, and the factors that currently limit translation of these systems beyond proof-of-concept studies.

## 2. Literature Search and Study Selection

This review was designed as a structured critical narrative review. A targeted narrative literature search was conducted by K.A.M. and D.G. using Scopus, Web of Science Core Collection, and Google Scholar. Search strings combined “methotrexate” with “cyclodextrin”, “β-cyclodextrin”, “hydroxypropyl-β-cyclodextrin”, “methyl-β-cyclodextrin”, “γ-cyclodextrin”, “inclusion complex”, “host-guest”, “hydrogel”, “nanocarrier”, “niosome”, “nanosponge”, “metal-organic framework”, “conjugate”, “capillary electrophoresis”, “solubility”, “release”, and “permeation”. Following peer review, a supplementary targeted search was performed to address CNS-directed delivery specifically, combining “methotrexate” and “cyclodextrin” with “central nervous system”, “CNS”, “brain”, “blood–brain barrier”, “BBB”, “glioma”, “glioblastoma”, “intracranial”, and “intrathecal”. No eligible experimental MTX–CD system specifically designed for CNS targeting or enhancement of MTX brain penetration was identified. Following peer review, an additional targeted search examined ternary complex strategies by combining “methotrexate” and “cyclodextrin” with “ternary complex”, “PVP”, “polyvinylpyrrolidone”, “PEG”, “polyethylene glycol”, “Poloxamer 407”, “HPMC”, “hydroxypropyl methylcellulose”, “PVA”, “polyvinyl alcohol”, and “hydrophilic polymer”. No additional MTX-specific study was identified in which these auxiliary excipients were mechanistically demonstrated to form or stabilize a discrete MTX–CD ternary molecular complex.

Searches were not restricted by publication date or language at the identification stage. Articles were eligible when they reported experimental, computational, analytical, pharmacokinetic, or biological data directly concerning MTX and a native, substituted, oligomeric, or polymeric cyclodextrin; a formulation based on a preformed MTX–CD complex; a cyclodextrin-containing carrier loaded with MTX; a covalent MTX–CD conjugate; or an analytical or safety application that depended on MTX–cyclodextrin interactions. General cyclodextrin reviews and clinical guidance documents were retained only for contextual statements and reference checking.

The literature collection initially comprised 68 full-text publications. Targeted searching of Scopus, Web of Science Core Collection, Google Scholar, publisher websites, and reference lists yielded eight additional full-text publications, giving 76 full texts that were assessed for eligibility. Five publications were excluded because they did not provide data directly relevant to an MTX–cyclodextrin interaction or to an MTX-containing cyclodextrin system. Accordingly, 71 full-text publications were retained for the thematic synthesis. One additional potentially relevant study published in 2024 was identified, but the full text could not be obtained and was therefore not used as a source of quantitative or mechanistic data. General cyclodextrin reviews, methodological papers, and clinical guidance documents were considered separately and were used only for contextual background. As the review was not prospectively designed as a systematic review, database-level identification and deduplication counts and formal inter-reviewer agreement statistics were not recorded.

Data were extracted into a structured evidence matrix that recorded cyclodextrin identity and architecture, the chemical relationship between MTX and the cyclodextrin component, preparation method, stoichiometry, association constant and units, methods used to support complexation, changes in solubility or stability, release and permeation behavior, pharmacokinetic findings, biological model, principal outcome, and study limitations. The central classification distinguished a classical inclusion complex, a formulation containing a preformed complex, a cyclodextrin-containing carrier without demonstrated MTX inclusion, and a covalent MTX–CD conjugate (Figure 1). Conflicting or incomplete evidence was retained as such rather than harmonized by assumption. For category assignment, the terminology used by the original authors was not considered sufficient by itself. A study was classified as a direct MTX–CD complex (Category A) when MTX was investigated with a chemically defined cyclodextrin host and molecular association was supported by solution-state evidence, including phase solubility analysis with an interpretable binding model, calorimetric binding measurements, NMR titration, or spatial NMR data. Where an isolated solid product was examined, DSC, XRD, FTIR, or related solid-state techniques were regarded as orthogonal supporting evidence rather than as stand-alone proof of cavity occupancy. Increased solubility, drug loading, disappearance of a melting transition, or spectral broadening alone were therefore not considered sufficient to demonstrate an inclusion complex.

A formulation was assigned to Category B when an MTX–CD complex satisfying the above criteria had been prepared and characterized before incorporation into a secondary dosage form or delivery platform. This designation describes the formulation history and does not imply that the intact molecular complex necessarily persists unchanged after incorporation into the final carrier. Category C was used when cyclodextrin constituted a structural or functional component of a carrier and MTX was loaded, adsorbed, encapsulated, or otherwise associated with the material without direct evidence of MTX occupancy of the cyclodextrin cavity. Systems in which the CD cavity was shown to bind another component of the formulation rather than MTX were also retained in Category C. Borderline systems were classified conservatively as Category C unless direct MTX–CD binding evidence justified placement in Category A or B. Category D was restricted to systems containing a covalent bond between MTX and the cyclodextrin scaffold.

The classification was mechanistic rather than a formal risk-of-bias grading system. Formal blinded duplicate classification and calculation of an inter-reviewer agreement coefficient were not part of the original review design; final assignments were based on the above predefined chemical and evidentiary criteria during construction of the evidence matrix.

## 3. Molecular Basis of Methotrexate Complexation

### 3.1. Methotrexate as a Cyclodextrin Guest

**Figure 2:**
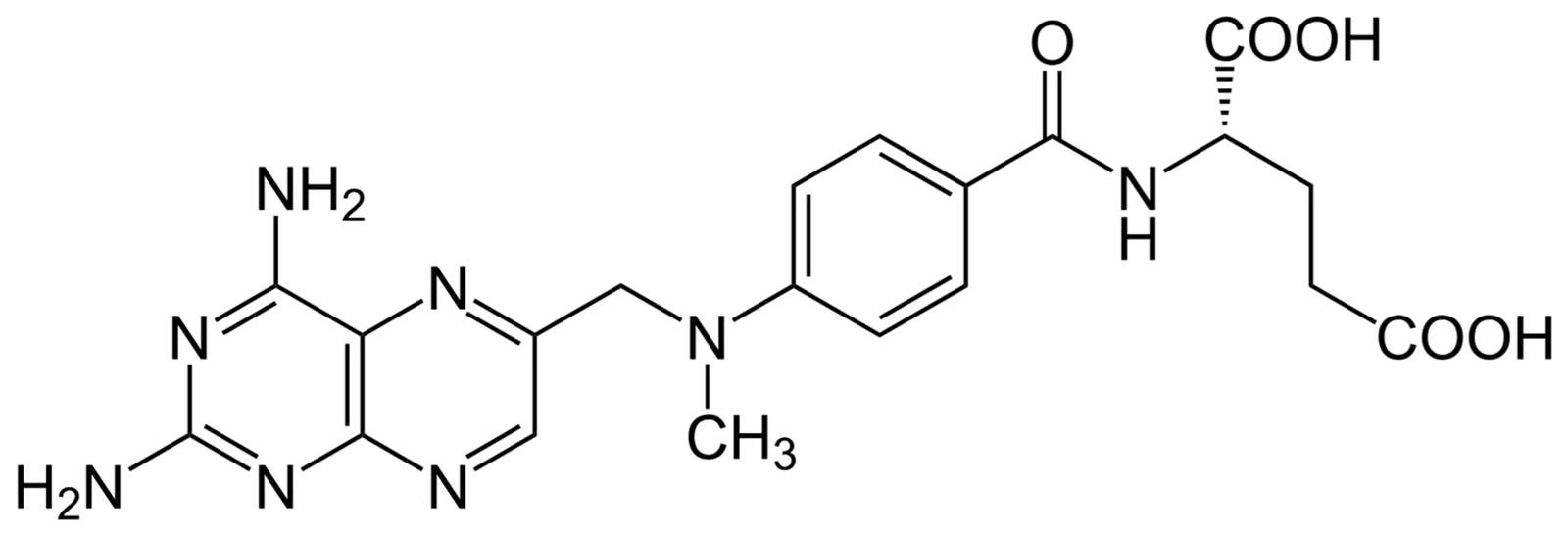
Chemical structure of MTX.

MTX is not a simple hydrophobe. Its pteridine ring, p-aminobenzoate unit, and glutamate residue create a flexible, ionizable molecule with several hydrogen bond donors and acceptors (Figure 2). Aromatic surfaces can enter a CD cavity and benefit from hydrophobic and dispersion interactions, while amino and carboxylate groups remain available for hydrogen bonding and electrostatic contacts at the rims. The balance changes with pH because protonation alters both conformation and the distribution of charge. An association constant reported for MTX is therefore conditional: it belongs to a defined temperature, ionic medium, pH, concentration range, and binding model. Throughout this review, direct MTX–CD association is distinguished from experimentally demonstrated cavity occupancy. Increased apparent solubility, dissolution, drug loading, or altered solid-state behavior alone is insufficient to establish inclusion, because these effects may also result from ionization, amorphization, adsorption, aggregation, or partitioning into non-CD domains. A direct reversible MTX–CD complex requires evidence of molecular association in a chemically defined host–guest system, such as an interpretable phase solubility equilibrium, calorimetry, or NMR titration. Confidence that MTX actually occupies the CD cavity is substantially increased by spatial evidence, particularly intermolecular ROESY/NOESY contacts between MTX protons and the internal H3/H5 protons of the cyclodextrin. DSC, PXRD, and FTIR are therefore treated as supportive orthogonal methods rather than stand-alone proof of inclusion. Accordingly, the term “molecular association” is used when MTX–CD binding is demonstrated without spatial localization, whereas “cavity occupancy” or “structurally supported inclusion” is reserved for systems with location-specific evidence. External association, adsorption, or partitioning remain alternative explanations when the evidence is limited to solubility changes, drug loading, 1D spectral perturbations, or altered solid-state behavior.

The foundational study by Singh and co-workers described an A_l_-type phase solubility relationship for MTX with β-CD, consistent with 1:1 association, and reported an apparent K_1:1_ of 469.5 M^−1^ [14]. This value should be regarded as a conditional parameter obtained under the experimental conditions of that study rather than as a universal reference constant. Subsequent independent measurements yielded values of the same general order of magnitude but not an exact reproduction of the original result. Pattarino et al. reported an apparent β-CD association constant of approximately 390 M^−1^, whereas ITC measurements by Aykaç et al. gave K = 0.54 ± 0.03 × 10^3^ M^−1^ for native β-CD at pH 7.2 and 25 °C [15,16]. Other experimental conditions have produced higher apparent values, further illustrating the conditional nature of MTX–β-CD association constants. Taken together, the available structural evidence most consistently implicates the p-aminobenzoate/phenyl region of MTX in CD recognition, whereas the polar glutamate residue is generally predicted to remain solvent-exposed. The position of the pteridine ring is less uniform and appears to depend on host architecture: some computational models permit deeper aromatic insertion, whereas the ROESY data for secondary-face β-CD dimers did not support deep pteridine inclusion. MTX should therefore not be represented by a single universal inclusion geometry.

The structural interpretation has also evolved since the original report. Singh et al. proposed, on the basis of molecular modelling and solid-state data, that an aromatic portion of MTX penetrates the β-CD cavity while the glutamate-containing region remains exposed [14]. Later 2D ROESY studies provided direct spatial evidence for MTX–β-CD contacts. Barbosa et al. observed correlations between aromatic MTX protons and the internal H3/H5 region of β-CD in the binary complex [17], while Silva et al. independently detected intermolecular contacts between MTX and internal cavity protons of native β-CD [18]. These later studies support cavity inclusion, although differences in experimental conditions and reported guest orientation indicate that no single static geometry should be regarded as uniquely established.

An early series of chromatographic studies by Cserháti and co-workers examined MTX among panels of anticancer agents interacting with hydroxypropyl-β-CD, carboxymethyl-β-CD, γ-CD, and α-CD [19,20,21,22]. Because these four publications originated from overlapping authors and closely related chromatographic approaches, they are treated here as a single historical evidence cluster rather than as four independent confirmations. Their principal value is qualitative: they established early that MTX interaction depends on cyclodextrin structure, but the methods were not designed to yield directly comparable modern thermodynamic parameters.

Stronger evidence for differences among the native cyclodextrins comes from later within-study comparisons. Pattarino et al. investigated α-, β-, and γ-CD within a common experimental framework using phase solubility and permeation measurements together with NMR and DSC. Under the reported conditions, no measurable complexation was detected with α-CD, γ-CD showed weak association (approximately 7 M^−1^), and β-CD showed substantially stronger association (approximately 390 M^−1^) [15]. Kritskiy et al. subsequently provided another direct comparison of native α-, β-, and γ-CD within a common study design [23]. These results support a qualitative preference for β-CD among the native hosts, but this should not be interpreted as a universal quantitative ranking independent of experimental conditions.

### 3.2. Substituted, Dimeric, and Polymeric Hosts

An early extension of this concept involved functionalization of β-CD with an epoxysuccinyl peptide designed to inhibit cathepsin B. Schaschke et al. reported that the resulting β-CD–peptide conjugate retained the ability to form an inclusion complex with MTX [24]. This 2000 study is relevant primarily as a historical demonstration that a targeting-related substituent can be appended to the CD scaffold without necessarily eliminating MTX recognition; it did not evaluate MTX release, pharmacokinetics, targeted delivery of the MTX-containing complex, or therapeutic efficacy.

Substitution of β-CD changes more than its aqueous solubility. It also reshapes the rims, modifies cavity hydration, and alters the conformational freedom of the glucose ring. Pattarino and colleagues found the largest association constant in their series for 2,6-dimethyl-β-CD, whereas 2,3-dimethylation and more extensive methylation did not produce an equivalent gain [15]. The conclusion is mechanistically important: the position of a substituent can matter more than the nominal degree of substitution. In a later within-study comparison of spray-dried complexes, the apparent association constants were 552.3 M^−1^ for native β-CD, 433.6 M^−1^ for HP-β-CD, 574.5 M^−1^ for M-β-CD, and 3114.7 M^−1^ for DM-β-CD [25]. Thus, DM-β-CD showed a markedly higher apparent affinity with the common experimental workflow and also provided the most favorable combination of dissolution, photostability, permeability-related behavior, and oral exposure.

Simple carbohydrate substituents offer a subtle way to tune recognition. At pH 7.4, UV–Vis titration with global fitting gave 1:1 association constants of 630 ± 5.5 M^−1^ for glucosyl-β-CD and 637 ± 8.6 M^−1^ for maltosyl-β-CD [26]. The authors compared these values with a previously reported native β-CD value of 389.7 ± 104.5 M^−1^; because that native host reference was obtained under different experimental conditions, the apparent approximately 1.6-fold increase should be interpreted qualitatively rather than as a strict within-study affinity ratio. Addition of the second glucose unit of maltose produced no further measurable gain. ^1^H and ^13^C NMR and modeling placed the phenyl ring in the cavity, with the appended sugar contributing contacts near the rim rather than enlarging the hydrophobic core [26].

Multivalent hosts can change both stoichiometry and geometry. Job plots supported a one dimer:two MTX stoichiometry for both secondary-face β-CD dimers. Reverse ITC in 50 mM sodium phosphate buffer at pH 7.2 and 25 °C gave site-specific association constants of 1.9 ± 0.2 × 10^3^ and 1.3 ± 0.3 × 10^3^ M^−1^ for dimers 1 and 2, respectively, compared with 0.54 ± 0.03 × 10^3^ M^−1^ for native β-CD [16]. Yet the 2D ROESY data did not support deep pteridine inclusion; they were more consistent with a shallow arrangement in which the aminobenzoate moiety caps the wider rim. A larger association constant, in other words, need not represent a more deeply included version of the same complex.

The comparison of monomeric, dimeric, and polymeric CDs makes the same point from another direction. A β-CD dimer showed strong cooperative 1:2 binding, whereas a β-polycyclodextrin displayed lower apparent affinity per accessible cavity, presumably because crosslinking generated a heterogeneous environment and made some cavities difficult to reach [23]. The overall constant of a 1:2 assembly was reported in M^−2^ and cannot be placed in a numerical ranking beside a 1:1 constant expressed in M^−1^. Such unit errors are surprisingly common in secondary summaries of the field.

Hybrid macromolecules introduce competing pockets. NOE NMR experiments on PAMAM dendrimer–CD conjugates showed that MTX could reside mainly within the dendrimer rather than the appended CD, depending on the cavity size and the architecture of the conjugate [27]. This study is a useful corrective to a common assumption: the presence of a CD moiety and successful drug loading do not, by themselves, demonstrate inclusion of that drug in the CD cavity.

### 3.3. Comparative Binding Data

The selected studies in Table 1 and Table 2 show a reproducible qualitative trend but also illustrate why absolute constants must be interpreted cautiously. Values were derived by phase solubility, calorimetry, capillary electrophoresis, NMR titration, or global fitting under different solution conditions. They describe related but not identical observables. The most informative comparisons are therefore those performed with one experimental design, where host chemistry is varied while pH, temperature, and analytical method remain constant. Accordingly, conclusions regarding the relative performance of native α-, β-, and γ-CD in this review are based primarily on direct within-study comparisons, whereas constants obtained across unrelated studies are used only to identify qualitative trends. Table 1 separates site-specific or 1:1 association constants expressed in M^−1^ from overall higher-order association parameters with different dimensionality. The latter should not be interpreted as numerically stronger or weaker solely on the basis of their absolute magnitude.

## 4. Preparation and Experimental Confirmation of Complexes

### 4.1. Preparation Methods and Solid-State Consequences

MTX–CD systems have been prepared by neutralization, kneading, freeze-drying, coprecipitation, spray drying, sonication, prolonged incubation in solution, and adsorption into crosslinked networks. These procedures are not interchangeable, and each introduces specific advantages and interpretive limitations. In the foundational Singh protocol, the so-called neutralization method was a pH-shift precipitation procedure. MTX was first dissolved in 0.1 M NaOH, after which an aqueous β-CD solution was added with stirring; 0.1 M HCl was then added dropwise with vigorous stirring until pH 7.5, at which point the product precipitated and was subsequently filtered, washed, and dried [14]. The alkaline stage maintains MTX in a more soluble ionized form and facilitates contact with β-CD, whereas lowering the pH reduces MTX solubility and permits isolation of the solid product. This procedure can efficiently generate a distinct solid, but the accompanying change in MTX ionization can also drive precipitation independently of cavity inclusion; consequently, precipitation itself should not be regarded as evidence of complex formation. Freeze-drying can preserve associations present in solution and produce rapidly dissolving porous solids, although extensive amorphization may confound interpretation of DSC and PXRD data. Kneading is simple, solvent-sparing, and experimentally accessible, but mixing may be heterogeneous and incomplete. Spray drying is rapid and potentially scalable and enables controlled within-study comparisons, but it simultaneously changes particle size and crystallinity, making it difficult to assign improved performance solely to molecular complexation. Sonication and prolonged solution incubation can improve molecular contact and dispersion, but neither procedure constitutes evidence of inclusion without independent structural characterization. Thus, differences among preparation methods may reflect inclusion, ionization, reduced crystallinity, particle size effects, or several mechanisms acting simultaneously.

The Singh study is instructive because it compares a physical mixture with kneaded, freeze-dried, and neutralization products. In XRD, the physical mixture largely showed the summed diffraction features of MTX and β-CD, whereas the neutralization product lost most prominent reflections and was markedly more amorphous [14]. DSC likewise distinguished the preparations: the β-CD water loss event, the MTX polymorphic transition exotherm, and the MTX melting endotherm disappeared in the neutralization product, whereas corresponding thermal events remained detectable or were shifted in the physical mixture. In IR spectroscopy, the physical mixture behaved largely as a summation spectrum, while the neutralization product showed a small shift of the MTX C–NH_2_ stretching band (1379 to 1383 cm^−1^), reduced intensity/resolution of MTX CH-bending vibrations, and suppression of β-CD bands near 1033 and 1156 cm^−1^. These changes support formation of a new and substantially altered solid phase, although they do not by themselves establish which part of MTX occupies the cyclodextrin cavity.

**Figure 3:**
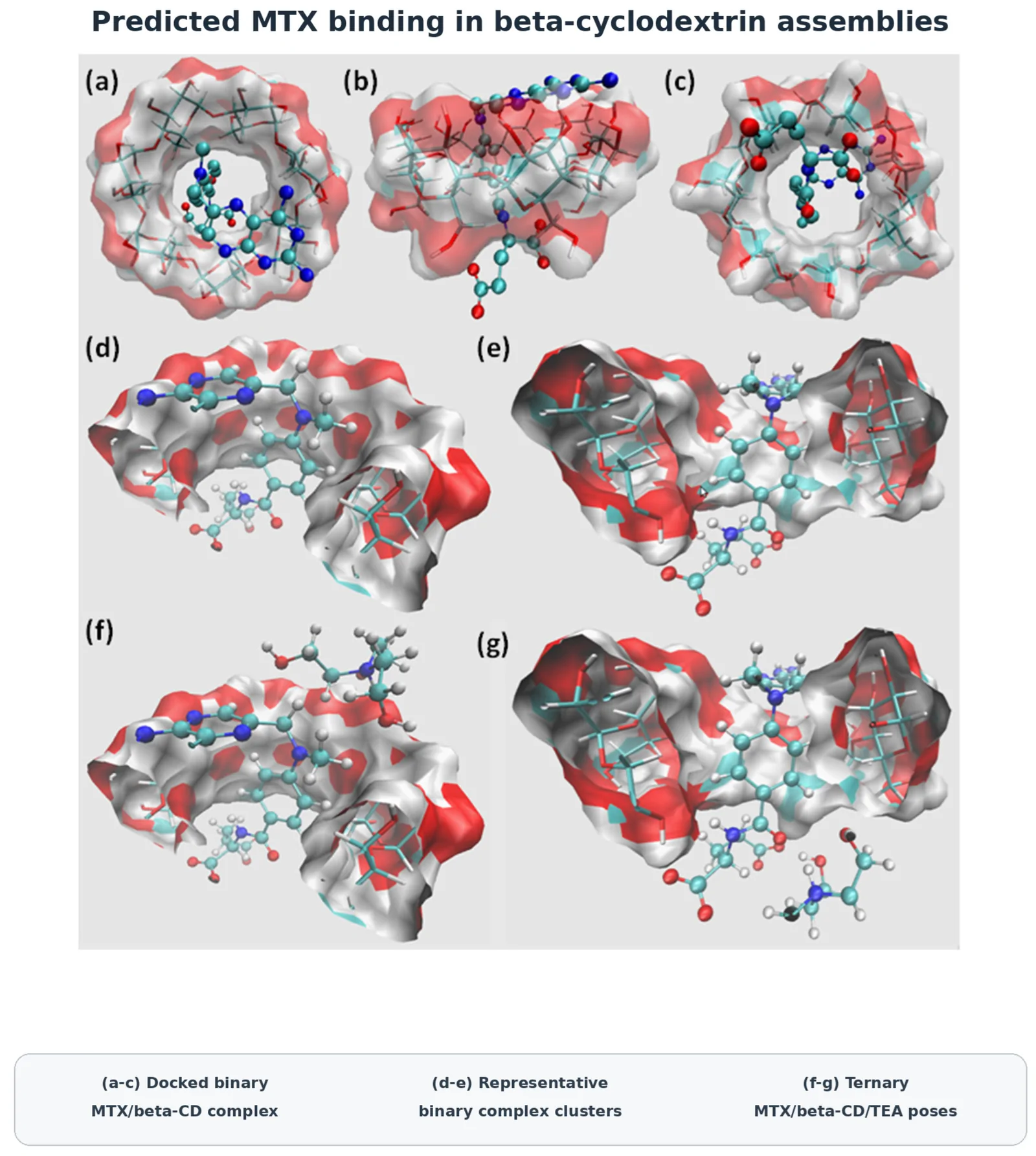
Predicted binding modes of MTX in binary and ternary β-cyclodextrin assemblies. Panels (**a**–**c**) show top, side, and rear views of docked MTX within β-CD; panels (**d**,**e**) show representative clusters of the binary MTX:β-CD system; and panels (**f**,**g**) show representative MTX:β-CD:triethanolamine poses. Adapted from Barbosa et al. [17] Spray drying has enabled useful within-study comparisons. Giri and colleagues prepared MTX complexes with β-CD, HP-β-CD, methyl-β-CD, and dimethyl-β-CD using a common process, then examined phase solubility, solid state, dissolution, photostability, Caco-2 transport, and pharmacokinetics [25]. Because the host was the principal controlled variable, the resulting rank order is more informative than a comparison assembled from unrelated experiments.

Ternary complexation adds another variable. In the MTX/β-CD/triethanolamine system, solution data supported a 1:1 MTX:β-CD association, whereas the isolated solid was prepared with a substantially larger molar excess of β-CD. Triethanolamine increased solubilization through a combination of ionization, electrostatic interactions, and host–guest binding [17]. The distinction between equilibrium stoichiometry and the molar recipe used to produce a formulation is essential; a 1:5 or 1:7 preparation ratio should not be reported as a discrete molecular complex without independent evidence. Representative binary and ternary arrangements are summarized in Figure 3.

Triethanolamine should not, however, be regarded as the only possible third component in cyclodextrin-assisted solubilization. In the broader pharmaceutical cyclodextrin literature, water-soluble polymers and surfactant-type excipients, including PVP, PEG, HPMC, PVA, and poloxamer-based systems, have been used to increase apparent complexation efficiency or solubilization [13]. Their effects may involve interactions with the external CD surface, stabilization of drug–CD aggregates, changes in hydration, or micellar-like solubilization rather than formation of a unique three-component inclusion geometry. Consequently, improved solubility in a drug/CD/polymer system should not by itself be interpreted as evidence of a discrete ternary molecular complex. In the MTX-specific literature examined here, the β-CD/triethanolamine study remains unusual because the influence of the third component was investigated directly using solution-state NMR, including ROESY, phase solubility analysis, and molecular modeling [17]. Polymer-assisted MTX–CD systems therefore represent a rational area for future systematic comparison, particularly using solution-state binding measurements and spatial NMR to distinguish enhanced complexation from nonspecific polymer- or surfactant-mediated solubilization.

Recent studies commonly prepare a complex in solution and then place it into a second carrier. The β-CD/MTX nanostructure reported by Hadi and co-workers was produced with sonication and examined experimentally and computationally [30]. Kumar and colleagues first precipitated a β-CD/MTX complex and subsequently incorporated it into an oil-in-water nanoemulsion thickened with Carbopol [29]. Such multistep protocols are attractive pharmaceutically, but they require analytical checkpoints after each stage because the complex may dissociate, repartition, or be displaced by surfactants and lipids.

### 4.2. What Constitutes Convincing Evidence?

No single analytical technique fully establishes an inclusion complex. Phase solubility analysis can suggest stoichiometry and yield an apparent constant, but the interpretation depends on intrinsic solubility, linearity, and the absence of higher-order aggregation. ITC provides stoichiometry and thermodynamic terms directly, yet model selection becomes difficult when several nonequivalent sites are present. One-dimensional NMR identifies perturbations in the guest environment; 2D ROESY or NOESY is more informative because it tests spatial proximity between guest protons and the internal H3/H5 protons of CD. Solid-state methods answer different questions: DSC and XRD reveal changes in crystallinity and phase behavior, while FTIR reports altered vibrational environments.

The evidentiary value of these techniques is greatest when chemically complementary methods are combined. Among the ten representative direct complex study groups summarized in Table 1, only one (10%) combined quantitative equilibrium or binding measurements, spatially informative 2D NMR, and solid-state characterization; six (60%) covered two of these three domains, whereas three (30%) were supported predominantly by a single domain. Thus, even within the comparatively well-characterized MTX–CD literature, fully orthogonal characterization remains uncommon.

The β-CD dimer study provides a particularly strong example of orthogonal solution-state evidence rather than of complete three-domain characterization. Job plots and ITC established a 1:2 dimer:MTX stoichiometry and quantified binding, while 2D ROESY showed that the large 1D chemical-shift changes did not imply deep pteridine inclusion. Instead, the aminobenzoate region was positioned close to the internal H3 proton and the wider secondary rim [16].

Mechanism-resolving controls are therefore as important as analytical sophistication. At minimum, experiments should compare free MTX, free CD, a physical mixture, and the isolated product. In hybrid carriers, an otherwise identical material without CD and, where feasible, a host with a blocked cavity provide more decisive tests. Biological experiments should also separate the effects of surfactant, oil, polymer, targeting ligand, and external stimulus from the effect of the molecular complex. Without those controls, the most defensible statement often concerns the performance of the complete formulation rather than the role of the CD cavity.

The evidentiary meaning of a given analytical technique also depends on the mechanistic category being investigated. The same observation may therefore carry very different weight in a direct molecular complex and in a heterogeneous carrier. Table 3 summarizes this distinction across the four categories used throughout this review. In particular, methods that are highly informative for Category A, such as quantitative binding measurements and spatial NMR, may become difficult to interpret in Category C because several non-CD binding domains coexist. Conversely, classical inclusion complex techniques are insufficient for Category D, where direct characterization of covalent bond formation, degree of substitution, hydrolysis, and released products is required.

## 5. Effects of Complexation on Methotrexate Properties

### 5.1. Solubility, Dissolution, and Solid-State Stability

The most consistent outcome of direct MTX–CD complexation is an increase in apparent solubility and dissolution rate. In the β-CD neutralization complex, faster dissolution was accompanied by a higher Cmax, an approximately two-fold increase in AUC, reduced degradation during storage, and improved survival in a BALB/c Ehrlich ascites carcinoma model [14]. At 15 mg kg^−1^ MTX equivalent, mean survival time increased from 32.3 days with free MTX to 36.7 days with the complex, corresponding to reported increases in life span of 70.0% and 93.16%, respectively. At 12.5 mg kg^−1^, the corresponding values were 28.5 versus 32.5 days and 50.0% versus 71.05%. The original authors reported *p* < 0.001 for the higher-dose comparisons, whereas the differences between free MTX and the complex at 2.5 and 7.5 mg kg^−1^ were not statistically significant. Free MTX at equivalent doses and a PBS vehicle group were included as controls, but no blank β-CD group was tested, and the number of animals per survival group was not reported. These historical findings support a possible formulation-related improvement in systemic exposure and antitumor efficacy, but they should be interpreted as proof-of-concept evidence obtained under 1997-era analytical, statistical, and reporting standards rather than as modern pharmacokinetic or efficacy validation. More recent within-study comparisons illustrate how strongly the magnitude of this benefit can depend on host chemistry and preparation method. In the spray-dried series reported by Giri et al., all four β-CD-based inclusion complexes dissolved more rapidly than free MTX, with 56.0%, 65.3%, 67.0%, and 81.7% of MTX released within 10 min from the HP-β-CD, β-CD, M-β-CD, and DM-β-CD complexes, respectively; release reached approximately 93–100% within 1 h [25]. The DM-β-CD formulation showed the largest effect and was reported to increase apparent aqueous solubility by approximately 2268-fold and in vitro drug release by approximately 1.89-fold relative to free MTX. These large formulation-level gains should not, however, be assigned exclusively to cavity binding, because spray drying also converted crystalline MTX into an amorphous form and reduced particle size, both of which can independently accelerate dissolution.

The magnitude of solubilization varies with host chemistry. The experimental/theoretical β-CD nanostructure increased apparent MTX solubility by roughly nine-fold, while the triethanolamine-containing ternary system produced an even larger concentration gain through combined ionization and inclusion [17,30]. Glucosyl and maltosyl substituents produced a more modest, chemically interpretable increase in affinity [26]. In hydrogels, however, changing the CD derivative simultaneously changes drug binding and network structure; the two effects cannot be separated from bulk release data alone [31].

### 5.2. Photostability and Chemical Protection

Photoprotection is one of the clearest practical advantages of several MTX–CD systems. In the spray-dried comparison of β-CD derivatives, free MTX exposed for 20 days to a light dose of 1.2 million lux·h at 25 °C/60% relative humidity showed a 36.8 ± 1.7% decrease in drug content together with visible color change. In contrast, the MTX–CD inclusion formulations showed only approximately 6.7 ± 1.9% loss of initial drug content under the same conditions [25]. This provides a quantitative example in which shielding of MTX within a host-rich microenvironment was associated with substantial protection against photolysis. A freeze-dried β-CD complex incorporated into SMEDDS similarly showed improved photostability together with an approximately ten-fold increase in aqueous solubility [32]. In the latter formulation, however, the protective effect cannot be assigned uniquely to the CD cavity, because the lipid phase, oxygen availability, and microenvironment within the final formulation may also influence photodegradation.

### 5.3. Free Drug, Permeability, and Bioavailability

**Figure 4:**
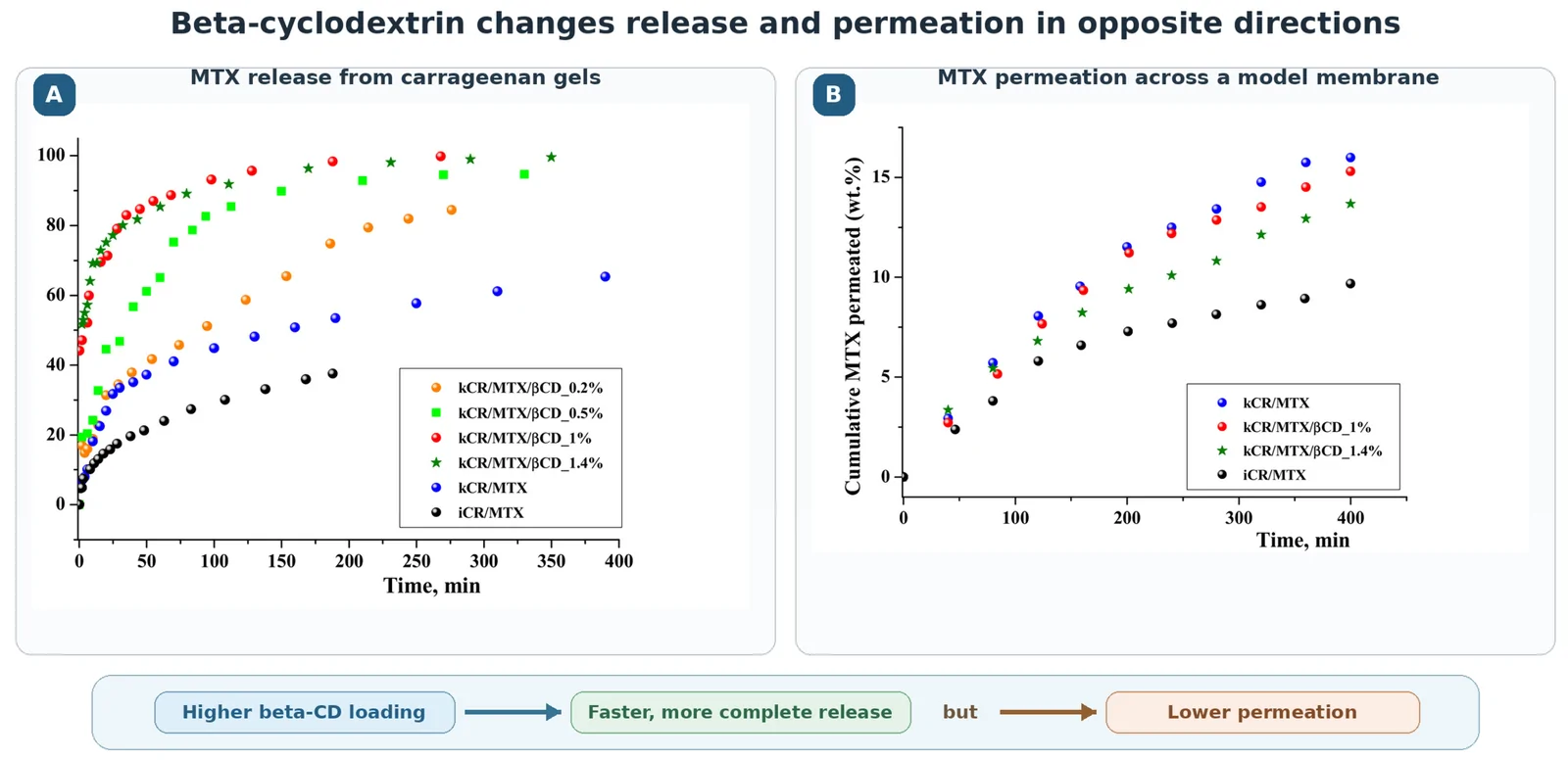
Opposing effects of β-cyclodextrin on MTX release and permeation from carrageenan hydrogels. (**A**) Cumulative MTX release from κ-carrageenan gels containing different β-CD concentrations at 37 °C and pH 7.4. (**B**) Cumulative MTX permeation from κ- and ι-carrageenan gels, showing reduced transport when β-CD was incorporated. Adapted, combined, and simplified from Figures 8 and 9 of Nikitina et al. [33] under the Creative Commons Attribution 4.0 License. Graphical redrawing was performed with limited AI assistance (Chat GPT, OpenAI, version 5.6); the final representation was verified by the authors against the original source.

Solubility and permeability are linked by an equilibrium that is easy to misread. CD increases total dissolved MTX, but a biological membrane primarily receives free drug. If the complex is large, charged, or slow to dissociate at the membrane surface, the free concentration can remain unchanged or even fall despite a higher total concentration [11]. This mechanism was visible in carrageenan hydrogels: β-CD increased the amount of MTX that could be loaded and released, yet membrane transport was lower than from the corresponding CD-free system [28,33]. The opposing release and permeation trends from the κ-carrageenan study are combined in Figure 4.

Oral pharmacokinetic studies provide several preclinical examples of increased systemic MTX exposure, although the extent to which the effect can be assigned specifically to cyclodextrin complexation varies among formulations. In the foundational 1997 β-CD study, oral administration of the neutralization complex increased the reported Cmax from 0.15 to 0.23 µg mL^−1^ and AUC from 11.73 to 24.57 µg·h mL^−1^, while Tmax remained 1 h [14]. This approximately two-fold AUC increase should be regarded as historical proof-of-concept because the analytical and pharmacokinetic reporting predates current standards.

A more recent rat study provides a substantially more detailed within-study comparison. Giri et al. evaluated free MTX and four spray-dried 1:1 complexes in groups of six rats. For the DM-β-CD complex, AUC increased from 1738.71 ± 294.65 to 3820.27 ± 424.27 h·ng mL^−1^ and Cmax from 265.63 ± 57.05 to 872.76 ± 62.19 ng mL^−1^; Tmax decreased from 1.13 ± 0.23 to 0.75 ± 0.29 h [25]. The corresponding relative increases in AUC and Cmax were approximately 2.20- and 3.29-fold, respectively. The HP-β-CD, native β-CD, and M-β-CD complexes also increased AUC, by approximately 1.84-, 1.74-, and 1.71-fold, respectively. In Caco-2 experiments, absorptive transport of free MTX and the DM-β-CD complex was similar, whereas the opposing efflux component was lower for the complex, further illustrating that the relationship between total solubilization and directional membrane transport is not linear.

A different magnitude of improvement was observed when a preformed β-CD complex was incorporated into a SMEDDS. This formulation increased oral MTX bioavailability by approximately 1.57-fold [32], but the contribution of complexation cannot be separated from lipid solubilization, microemulsification, and surfactant effects. The largest reported pharmacokinetic change occurred with the γ-CD metal–organic framework. Following oral administration in rats, Cmax increased from 1.3 to 3.6 µg mL^−1^, AUC0–7 from 1.0 to 12.8 µg·h mL^−1^, and elimination half-life from 1.5 to 4.5 h, while Tmax shifted from 1 to 2 h [34]. The authors reported an increase in apparent absolute bioavailability from approximately 1% to 16%. These unusually large changes should be attributed to the γ-CD-MOF architecture as a whole—including dissolution, porous framework retention, and altered permeability—rather than to γ-CD cavity inclusion alone.

Taken together, the animal studies show that MTX–CD-containing formulations can substantially modify systemic exposure, but they do not establish a uniform pharmacokinetic effect of complexation. No human pharmacokinetic study of an MTX–CD therapeutic formulation was identified in the present search, and translation therefore remains dependent on comparative studies that measure free and total MTX, complex dissociation, tissue exposure, and safety in addition to conventional plasma pharmacokinetics.

The same solubility–permeability distinction would be especially relevant to CNS delivery; however, no MTX–CD formulation specifically designed to cross the blood–brain barrier or increase brain exposure was identified in the present search. Thus, it remains unknown whether the increased aqueous availability achievable through MTX–CD complexation could be translated into greater CNS exposure. A strongly bound complex might instead reduce the freely available MTX concentration at the endothelial interface unless sufficiently rapid dissociation occurs near the blood–brain barrier.

### 5.4. Release and Biological Activity

**Figure 5:**
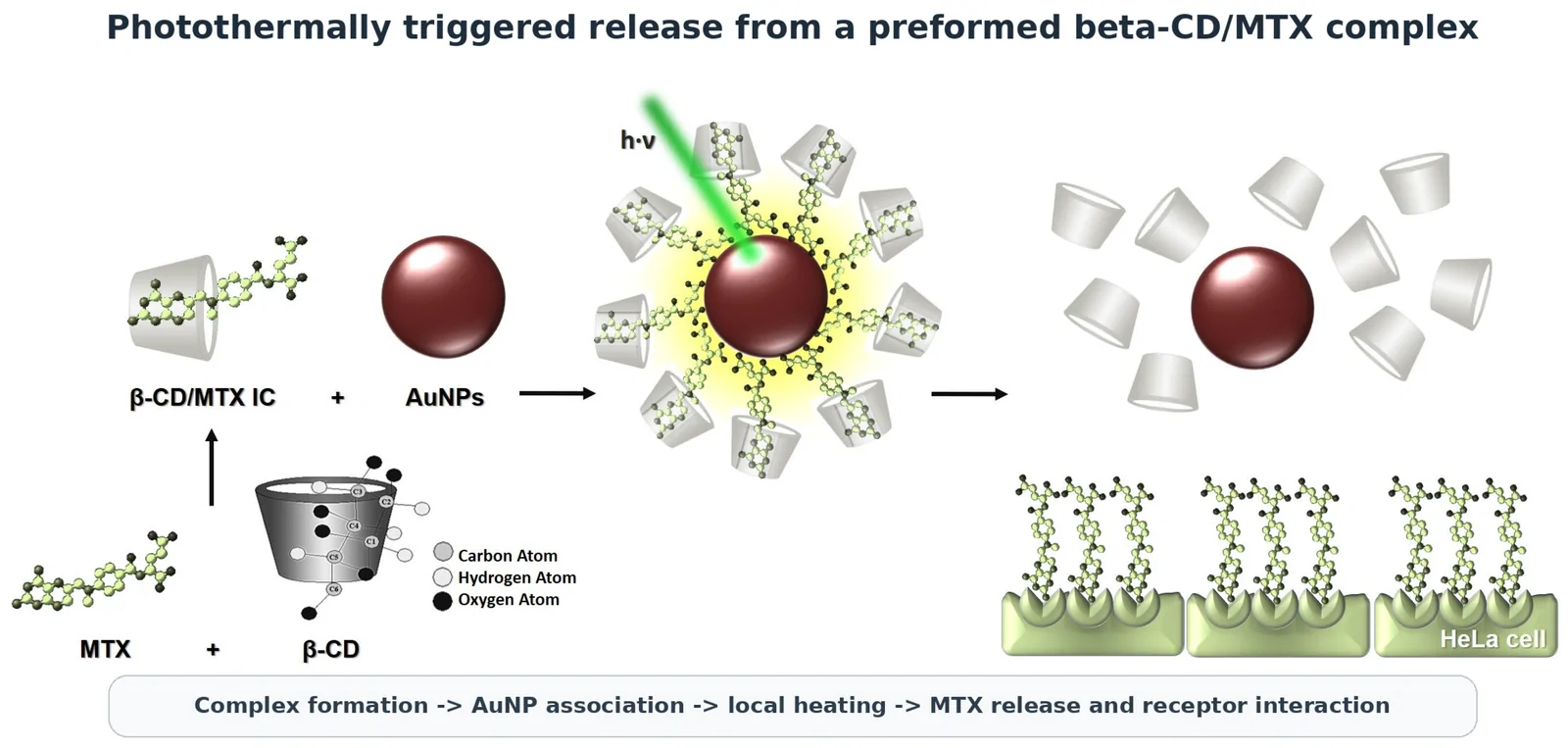
Photothermally triggered MTX release from a preformed β-CD/MTX inclusion complex associated with gold nanoparticles. Irradiation is converted into local heat, promoting dissociation of the complex and release of MTX for interaction with folate receptors on HeLa cells. Adapted from Silva et al. [18].

Complexation can either accelerate release by increasing aqueous availability or retard it by retaining MTX within a host-rich matrix. Gelatin hydrogels crosslinked with β-CD increased drug loading and markedly reduced burst release compared with a control hydrogel [35]. Carrageenan systems released MTX rapidly once sufficient CD was present, but permeability decreased, as noted above. In photothermal β-CD/MTX-gold nanoparticle assemblies, irradiation released part of the complexed drug and restored cytotoxic activity that had been attenuated while MTX was shielded [18]. The sequence of complex formation, gold nanoparticle association, photothermal heating, and drug release is illustrated in Figure 5.

Biological improvement should be interpreted at the tested level. A more active cell formulation may reflect increased uptake, slower extracellular loss, membrane perturbation by excipients, or an external trigger rather than a different intrinsic potency of complexed MTX. The 2026 topical nanogel is a useful example. It combined a β-CD complex, nanoemulsion droplets, surfactant, co-surfactant, oil, and a Carbopol matrix; the formulation improved release, porcine skin permeation, and outcomes in adjuvant-induced arthritis [29]. The data support the nanogel, but they do not isolate β-CD complexation as the sole causal factor.

Thus, the principal demonstrated advantages of MTX–CD technology span different levels of evidence: direct complexes can improve solubility, dissolution, and photostability; incorporation into secondary carriers can further alter retention, release, permeability, and systemic exposure; and biological efficacy improvements are currently demonstrated mainly in cell and rodent models. The increasingly large effects observed as formulation complexity increases should therefore be interpreted together with a decreasing ability to attribute the outcome specifically to MTX–CD cavity binding.

## 6. Direct Use and Formulations Based on a Preformed MTX–CD Complex

### 6.1. Direct Administration of Isolated MTX–CD Complexes

Not all pharmaceutically evaluated MTX–CD systems require a secondary carrier. In several studies, the isolated molecular complex itself constituted the administered formulation. In the foundational study by Singh et al., the β-CD neutralization complex was isolated as a solid and subsequently dispersed in phosphate-buffered saline for oral administration. In the pharmacokinetic experiment, the complex was administered directly at an MTX-equivalent dose of 5 mg kg^−1^, without incorporation into a lipid, polymeric, or particulate carrier [14]. The same directly administered complex was also used in the Ehrlich ascites carcinoma survival experiment. Thus, although the study did not develop a finished clinical dosage form, it represents an early example in which the MTX–CD complex itself, rather than a secondary delivery platform, was the pharmaceutical intervention.

A more recent example was provided by Giri et al., who prepared spray-dried 1:1 complexes of MTX with β-CD, HP-β-CD, M-β-CD, and DM-β-CD [25]. The isolated powders were filled into size 0 hard gelatin capsules for dissolution testing, providing a direct oral solid dosage form concept. For the rat pharmacokinetic study, the same isolated complexes were administered by oral gavage as 1 mL aqueous suspensions equivalent to 20 mg kg^−1^ MTX. No secondary lipid, polymeric, or nanoparticulate carrier was used. Among the tested hosts, the DM-β-CD complex produced the largest increase in systemic exposure, with approximately 2.20-fold higher AUC and 3.29-fold higher Cmax than free MTX. These data demonstrate that an isolated MTX–CD complex can itself function as a preclinical oral formulation rather than solely as an intermediate for subsequent carrier incorporation.

Despite the pharmaceutical relevance of this simpler strategy, we identified no study in which a directly characterized MTX–CD inclusion complex was developed as a simple injectable solution and evaluated without an additional carrier. Reported injectable CD-containing MTX systems instead employ hydrogels or other multicomponent architectures in which cyclodextrin also contributes to carrier assembly. Direct parenteral MTX–CD formulations therefore remain an underexplored area, and future development would require consideration of cyclodextrin derivative selection, solution stability, osmolality, free MTX fraction, renal handling, and compatibility with parenteral administration.

### 6.2. Vesicular and Lipid-Based Systems

Placing a preformed complex into a second carrier is one of the oldest strategies in the MTX–CD literature. Two closely related studies from the same research program examined the incorporation of preformed MTX–CD complexes into niosomes. In the earlier study, an HP-β-CD–MTX complex increased apparent niosomal entrapment from 67% for non-complexed MTX to 89%, slowed release, improved storage stability, and increased life-span response in tumor-bearing BALB/c mice [36]. A subsequent study from the same institution used native β-CD and reported 84% entrapment for the MTX–β-CD complex versus 67% for non-complexed MTX, together with improved stability and tumor growth outcomes in C57BL/6J mice bearing B16F1 melanomas [37]. The studies used different CD hosts and distinct in vivo models and therefore represent separate biological experiments; however, their closely related formulation methodology and shared research provenance mean that they should be regarded as related evidence rather than as two independent replications. The original reports do not provide sufficient batch-level information to establish independence of all formulation controls. Taken together, they provide early proof of concept that precomplexation can increase the amount of MTX retained within the aqueous compartment of nonionic surfactant vesicles, while the vesicle membrane provides an additional diffusion barrier.

The SMEDDS formulation extended the same logic to oral delivery. A freeze-dried β-CD complex was combined with an oil, surfactant, and co-solvent mixture that dispersed into small droplets on contact with gastrointestinal fluid. The reported formulation increased MTX solubility by approximately an order of magnitude, reduced photodegradation, and raised oral bioavailability [32]. The approach is pharmaceutically credible, but the unusual high CD ratio used in preparation should not be confused with molecular stoichiometry.

### 6.3. Hydrogels, Porous Hosts, and Hybrid Particles

A preformed complex can also be embedded in a matrix intended for local or sustained delivery. β-CD-crosslinked gelatin hydrogels used the host both as a chemical crosslinker and as a reversible MTX binding site [35]. Iota- and kappa-carrageenan gels used β-CD to raise drug loading and tune release, although the same complexation reduced membrane transport in some conditions [28,33]. More recent iota-carrageenan work compared native, hydroxypropylated, and amino-substituted β-CDs, showing that host chemistry also changes the rheology of the gel network [31].

Hybrid inorganic systems add externally controllable or high-capacity domains. In the β-CD/MTX-gold nanoparticle system, the molecular complex reduced free drug activity until green light irradiation induced photothermal release [18]. γ-CD metal–organic frameworks offered a porous crystalline network with substantial loading and striking pharmacokinetic effects in rats [34].

The topical β-CD/MTX nanoemulsion gel for rheumatoid arthritis is the most recent example of a clearly preformed complex carried into a secondary dosage form. The complex was supported by phase solubility analysis, thermal methods, XRD, and 1H NMR, then incorporated into a chaulmoogra oil nanoemulsion and a Carbopol gel. Release reached about 78% over 24 h, and ex vivo permeation exceeded that of a conventional gel. In the CFA-induced arthritis model, therapeutic response was assessed using a semi-quantitative 0–4 clinical arthritis score based on swelling and erythema, serial hind paw thickness, body weight, and day 22 radiographic evaluation of joint space integrity and bone erosion; SGOT, SGPT, and ALP were also measured as hepatic safety markers [29]. The nanogel reduced the clinical arthritis score toward 1 and paw thickness toward near-normal values by day 22 and showed improved radiographic joint architecture compared with the disease control and conventional MTX gel. However, histopathological scoring and direct functional measures such as gait, weight-bearing, or joint mobility were not included. The small group size, 22 day observation period, and multicomponent formulation further limit translational and mechanistic attribution.

Despite measurable improvements in formulation performance, the systems discussed in this section remain entirely preclinical. No human pharmacokinetic or clinical efficacy study of an MTX–CD formulation was identified. The available animal data show that improved physicochemical properties can translate into greater systemic exposure: the foundational β-CD complex approximately doubled AUC, the spray-dried DM-β-CD complex increased AUC and Cmax by approximately 2.20- and 3.29-fold, respectively, and the β-CD/SMEDDS formulation increased relative bioavailability by approximately 1.57-fold [14,25,32]. However, these findings do not establish reduced MTX toxicity. In the Giri et al. study, free MTX and the DM-β-CD complex showed no substantial cytotoxicity in Caco-2 cells up to 200 μM, but this experiment was designed primarily to support permeability testing and cannot be interpreted as evidence of improved systemic safety [25].

The more complex formulations introduce additional uncertainties. The large pharmacokinetic effect of the γ-CD metal–organic framework reflects the porous framework as a whole rather than γ-CD inclusion alone [34]. The historical niosome studies reported improved antitumor outcomes but did not provide contemporary toxicological characterization [36,37]. The photothermal gold system remained an in vitro proof of concept [18], whereas the topical nanogel included short-term hepatic biochemical markers but lacked chronic toxicology, histopathology, systemic pharmacokinetics, and long-term dermal assessment [29]. Thus, efficacy improvement and safety improvement should be considered separate endpoints.

The fate of the molecular complex after administration also remains incompletely resolved. No study identified here directly tracked intact MTX–CD complexes in vivo or measured the site and kinetics of their dissociation. For orally administered reversible complexes, the working model is a dynamic equilibrium in which CD increases the dissolved MTX pool but uncomplexed MTX is released before or near the epithelial membrane and becomes available for absorption [11]. Secondary carriers introduce additional release mechanisms, including diffusion from vesicles or gels, partitioning into lipid phases, carrier disassembly, and stimulus-triggered release. Clinically relevant comparisons with conventional oral, subcutaneous, or intravenous MTX at matched systemic exposure remain absent (Table 4).

Values are reported as presented in the source studies. Improvements observed for the complete formulation should not be attributed to the CD cavity alone, unless mechanism-resolving controls were included.

## 7. CD-Containing Carriers Without Complete Proof of MTX Inclusion

### 7.1. Dendritic, Polymeric, and Multimodal Platforms

A second, much larger body of literature uses CD as part of a carrier but does not always establish that MTX is the guest occupying its cavity. These systems are valuable, yet they answer a different question from classical inclusion chemistry. Porphyrin–CD conjugates, for example, combined drug binding, tumor accumulation, optical imaging, and photodynamic therapy [38]. Chitosan/CD nanoparticles were designed to co-load hydrophilic and hydrophobic agents [39]. In both cases, the pharmacological outcome reflects a multifunctional material rather than an isolated MTX–CD equilibrium.

Dendritic constructs exploit the contrast between a CD core and a cationic, highly branched exterior. Star-shaped β-CD-PAMAM polymers were able to bind siRNA through the dendrons while retaining MTX in or near the central CD domain [40]. Poly(L-lysine) dendrons attached to CD were developed for combined gene and drug delivery [41], and click chemistry produced primary-face dendritic and biodendrimeric β-CD derivatives with improved loading capacity [42,43]. These architectures contain several hydrophobic, ionic, and hydrogen-bonding regions; direct NMR localization is needed before MTX is assigned to the CD cavity.

A more conventional polymeric example is the PLGA-β-CD nanoparticle. MTX-loaded particles altered release and cytotoxicity in T47D breast cancer cells [44]. The contribution of β-CD could include drug binding, changes in polymer hydration, or effects on particle morphology. A formulation control made from otherwise identical PLGA without β-CD is therefore more informative than a comparison with free MTX alone.

### 7.2. Carbon, Magnetic, Gold, and Biomimetic Carriers

Graphene oxide modified with a stimulus-responsive polymer brush bearing pendant β-CD released MTX in response to pH and temperature [45]. Iron oxide and magnetic supramolecular carriers were used for single or combined delivery of MTX and tamoxifen, sometimes with a cascade architecture or apoptosis modeling [46,47,48]. The magnetic core adds imaging, targeting, and heating possibilities, but it also makes the origin of improved uptake difficult to disentangle. Reported “host–guest” behavior should be tied to direct binding data rather than inferred from release alone.

Gold glyconanoparticles bearing β-CD and carbohydrate ligands illustrate another design logic: the sugar surface can recognize lectins, while the CD layer provides drug-loading capacity [49]. Recent biomimetic systems have moved beyond cancer. β-CD-containing nanoparticles were coated with cell membranes or combined with dopamine-modified polycyclodextrins to influence cholesterol handling and deliver MTX in models of atherosclerosis [50,51]. These studies broaden the therapeutic context of MTX–CD materials, but the anti-atherosclerotic effect includes membrane camouflage, cholesterol transport, and immunomodulation in addition to drug release.

### 7.3. Hydrogels, Organogels, and Nanosponges

CD can serve as a physical junction, a crosslinking component, or a local solubilizing domain in gels. A β-CD supramolecular organogel enhanced the apparent anticancer effect of MTX [52]. Carboxymethylcellulose/β-CD/chitosan and xanthan/HP-β-CD hydrogels combined drug release with magnetic or antibacterial functionality [53,54]. A theranostic β-CD hydrogel containing Fe_3_O_4_ and Bi_2_O_3_ added magnetic responsiveness and imaging-related components [55]. In each case, CD participates in the matrix, and the material should not be described as a pure MTX inclusion complex unless guest occupancy is demonstrated independently.

The injectable polymerized β-CD/PEG-cholesterol hydrogel is particularly revealing. Its network forms because cholesterol end groups enter polymerized β-CD cavities. MTX and 5-fluorouracil are then distributed through the hydrated network, with sustained release and increased exposure in a rat breast cancer model [56,57]. The proven host–guest pair is CD–cholesterol, not necessarily CD-MTX. Describing the system accurately does not diminish its value; it clarifies which supramolecular interaction is responsible for gelation.

β-CD nanosponges represent a different network in which multiple cavities and crosslinker-derived domains can retain MTX. A site-retentive β-CD nanosponge nanogel was evaluated using RAW264.7 macrophage cell viability and scratch–migration assays and subsequently in a Freund’s complete adjuvant-induced inflammation model, in which superior local anti-arthritic activity was reported [58]. The study supports prolonged intra-articular retention and local pharmacological activity, but a validated chronic joint function endpoint such as gait, weight-bearing, or mobility testing was not identified. As with other composite gels, a factorial comparison of the nanosponge, gel base, and complete formulation would be needed to assign the observed benefit to one component.

### 7.4. Stimulus-Responsive and Combination Delivery Systems

Stimulus-responsive carriers make use of host–guest chemistry to build a structure that can be dismantled by a change in pH, light, temperature, redox state, or ultrasound. Poly(α-CD) and an acetal-modified β-CD-azobenzene derivative assembled into nanoparticles through α-CD/azobenzene inclusion. MTX served as a model payload and was added during nanoparticle assembly, after which the dispersion was dialyzed to remove unretained material. The reported drug-loading content (2.09%) and entrapment efficiency (21.3%) were calculated from the total MTX retained by the nanoparticle formulation and therefore represent whole particle loading metrics rather than measures of cyclodextrin cavity occupancy [59]. The loaded nanoparticles showed rapid MTX release at pH 5 and burst release during UV irradiation. Importantly, the experimentally demonstrated host–guest interaction responsible for carrier assembly was between poly(α-CD) and the azobenzene moiety of β-CD-Azo-Ace; the study did not spatially localize MTX within the particles or demonstrate its inclusion in either cyclodextrin cavity.

An aminated CD/sodium dodecyl sulfate nanocarrier was designed to protect orally administered MTX in the gastrointestinal tract and release it in response to pH and temperature. The formulation improved uptake and apoptosis in colon cancer cells, but it contained several ionic and hydrophobic domains and was not evaluated in vivo [60]. An ultrasound-augmented polyoxometalate/β-CD assembly was evaluated in a collagen-induced arthritis mouse model [61]. In addition to joint accumulation and overall disease attenuation, the study examined mechanistic endpoints including ROS scavenging and O_2_ generation, regulation of HIF-1α-associated hypoxia, and synovial macrophage polarization from an M1-like toward an M2-like phenotype. These readouts provide substantially greater mechanistic depth than paw swelling measurements alone, but they do not substitute for chronic functional assessment of locomotion, load bearing, or joint mobility. Moreover, the biological effect remains attributable to the combined POM, CD assembly, MTX, and ultrasound intervention rather than to CD complexation alone.

Carbohydrate decoration and combination therapy provide further layers of function. Maltose-clustered and glucose-functionalized β-CD carriers increased drug-loading or cellular interactions [62,63]. A carbonic-anhydrase-IX-targeting, glutathione-responsive host–guest amphiphile was designed for tumor-selective delivery [64]. A ternary β-CD/curcumin/MTX compound and β-CD/alginate nanocomposites illustrate the growing interest in multidrug and statistical formulation design [65,66]. Cationic CD-coated magnetic nanoparticles likewise delivered MTX to Saos-2 osteosarcoma cells in a pH-responsive system [67].

A β-CD-grafted bacterial cellulose nanocrystal carrier published in 2026 combines accessible CD cavities with pH-responsive DMAEMA and thermoresponsive PNIPAAm. MTX and curcumin were co-loaded, and the dual formulation showed an MTX entrapment efficiency of 64.5%, enhanced release at pH 5.8 and 40^°^ C, and strong synergistic cytotoxicity in MCF-7 cells [68]. The authors quantified accessible β-CD sites with phenolphthalein, but this proves the availability of cavities rather than the location of MTX.

Taken together, most systems discussed in this section should not be regarded simply as incompletely characterized versions of direct MTX–CD complexes. Among the 11 representative Category C architectures summarized in Table 5, only 2 (18%) were judged to be plausibly reclassifiable at the system level as Category A or B if direct MTX–CD binding and, where relevant, precomplex formation were demonstrated. In six cases (55%), MTX occupancy of a CD cavity remains chemically plausible, but demonstration of such an interaction would establish only one molecular contribution within a larger carrier and would not by itself justify reclassification of the complete formulation. Three systems (27%) are intrinsically carrier-level architectures in the present design, because cyclodextrin primarily participates in carrier assembly or interacts with another structural guest. Accordingly, approximately 82% of these representative systems would remain within Category C even after more complete molecular characterization. This distinction indicates that much of the diversity of the recent MTX–CD literature reflects innovation in CD-containing carrier architecture rather than unverified classical inclusion complexes. The translational evidence is even more limited for these Category C carriers. None of the representative systems has progressed to human pharmacokinetic or clinical evaluation as an MTX formulation. Toxicological assessment is commonly restricted to short-term cell viability, apoptosis, or efficacy experiments, and these endpoints cannot establish the safety of the carrier itself. In particular, polymerized, crosslinked, amino-substituted, magnetic, graphene-based, and grafted cyclodextrin architectures introduce chemical components whose degradation, tissue persistence, immunogenicity, membrane effects, and renal elimination may differ substantially from those of soluble pharmaceutical cyclodextrins. Their safety therefore cannot be inferred from the clinical use of HP-β-CD or SBE-β-CD as excipients.

The fate of MTX is also more difficult to assign in Category C systems because the drug may exchange among CD cavities, polymer domains, lipid or surfactant regions, particle surfaces, and the surrounding aqueous phase. Consequently, release from the complete carrier should not automatically be interpreted as dissociation of an MTX–CD inclusion complex. Most studies compare the complete carrier with free MTX, whereas factorial controls and clinically relevant conventional MTX formulations are uncommon. Translation will require exposure-matched comparisons, tissue distribution and carrier clearance studies, and repeated-dose toxicology in addition to conventional efficacy measurements.

## 8. Covalent MTX–CD Conjugates

### 8.1. Colon-Directed Ester Prodrugs

Covalent attachment changes the scientific question from reversible molecular recognition to prodrug chemistry. Gupta and colleagues esterified MTX with α- and γ-CD, aiming to mask the carboxylic acid groups responsible for direct gastrointestinal irritation. The conjugates showed negligible release in simulated gastric and intestinal fluids but underwent extensive hydrolysis in rat fecal material. In the ulcerogenicity experiment, free MTX administered at 12.5 mg kg^−1^ produced a reported ulcer index of 63 ± 0.94, whereas no detectable lesions (“Nil”) were reported for the α-CD ester SRD1 at 38.7 mg kg^−1^, and the γ-CD ester SRD2 at 49.6 mg kg^−1^ produced an ulcer index of 14.6 ± 0.82 [69]. The original table describes these values as averages of six readings and reports *p* < 0.05, but it does not clearly state whether these readings represent six independent animals per group, identify the statistical test used, or specify the comparisons to which the reported *p*-value applies. The apparent reduction in ulcerogenicity should therefore be interpreted as preliminary preclinical evidence rather than as a quantitatively robust demonstration of improved gastrointestinal safety. These preliminary findings are consistent with the rationale for masking MTX carboxyl groups in a colon-directed prodrug, but they do not establish a clinically relevant reduction in gastrointestinal toxicity and do not describe an inclusion equilibrium.

The study also illustrates the analytical demands of a conjugate. A reliable prodrug assessment requires control over which MTX carboxyl group is esterified, the degree of substitution on CD, residual free drug, stereochemical integrity, hydrolysis kinetics, and the identity of released products. Older synthetic reports often relied on IR and proton NMR without the mass spectrometric and chromatographic resolution expected today.

### 8.2. Enzymatic and Targeted Conjugates

Noro and co-workers used ultrasound-assisted enzymatic synthesis to attach one or more MTX residues to α-, β-, and γ-CD. Ultrasound increased conversion and shortened the process, and MALDI-TOF/NMR supported formation of multivalent conjugates [70]. The work is a synthesis study rather than a delivery study: it does not establish drug release rates, biological stability, or whether the conjugated MTX retains activity after enzymatic cleavage.

A biotin-functionalized antitumor construct combined a covalent MTX-β-CD conjugate with a biotin–adamantane partner. The host–guest interaction in the final assembly is β-CD binding adamantane; MTX is covalently attached and should not be called the guest [71]. This distinction matters because release is governed by bond cleavage, while assembly and targeting depend on the separate adamantane/CD equilibrium.

For future conjugates, pharmacological evaluation should include the intact construct, the corresponding physical mixture, released MTX, and a noncleavable analog. Such comparisons would reveal whether activity comes from the prodrug, premature free drug contamination, or intracellular hydrolysis. They would also allow covalent systems to be compared on clinically relevant exposure rather than on a nominal “loading” that cannot be equated with association capacity.

### 8.3. Safety and Translational Status of Covalent Systems

Covalent MTX–CD systems provide the clearest suggestion within the reviewed literature that CD-based modification may alter a toxicity-related endpoint, but the evidence remains preliminary. In the Gupta et al. study, the α-CD ester produced no detectable gastric lesions, and the γ-CD ester markedly reduced the reported ulcer index relative to free MTX [69]. However, this experiment had limited statistical reporting, did not establish a clinically relevant gastrointestinal safety benefit, and represents a covalent prodrug strategy rather than reversible cavity complexation. It therefore should not be cited as evidence that MTX–CD inclusion itself reduces MTX toxicity.

The later covalent systems provide even less translational safety information. The enzymatically synthesized α-, β-, and γ-CD conjugates were characterized chemically but were not evaluated for pharmacokinetics, repeated-dose toxicity, or biological activity following cleavage [70]. Likewise, the biotin–adamantane construct demonstrates a sophisticated targeting architecture but does not establish human exposure, systemic safety, or therapeutic superiority over conventional MTX [71]. For covalent systems, translation will require quantitative identification of circulating intact conjugate and released MTX, cleavage kinetics in relevant tissues, characterization of all released CD-containing products, and comparison of efficacy and toxicity at equivalent MTX exposure. No human pharmacokinetic or clinical study of a covalent MTX–CD conjugate was identified.

## 9. Analytical Applications and Safety

### 9.1. Chiral and Impurity Analysis

Cyclodextrin-MTX recognition has an important analytical life independent of drug delivery. Therapeutic MTX is the L-enantiomer, while D-MTX is a distomer with different pharmacokinetics and is relevant to quality control. HP-β-CD-modified capillary electrophoresis separated the enantiomers in tablets and injections with recoveries above 93% [72]. In CD-modified micellar electrokinetic chromatography, native β-CD was essential for resolving D-/L-MTX and achiral impurities; the optimized system contained 100 mM SDS, 45 mM β-CD, and 25% methanol and achieved recoveries of 93–106% [73].

Other formats exploited the same stereoselective interaction. Quenched phosphorescence provided sensitive detection after HP-β-CD-mediated electrokinetic separation [74]. A carbon paste electrode modified with HP-β-CD accumulated MTX enantiomers differently and enabled voltametric assessment of enantiomeric purity [75]. In these methods, CD is a chiral pseudostationary phase or recognition layer, not a pharmaceutical carrier.

These studies should be viewed primarily as legacy MTX-specific demonstrations of CD-mediated chiral recognition rather than as a survey of current analytical practice. The earliest CE, MEKC, and voltametric approaches were reported in 2002–2006 [72,73,75], followed by the phosphorescence-coupled electrokinetic method in 2011 [74]. Our search did not identify a post-2015 MTX-specific CD-based enantioseparation method directly replacing these approaches. This absence should not be interpreted as obsolescence of CD-assisted chiral electrophoresis itself: the broader field remains active, with contemporary work emphasizing structurally defined cyclodextrin selectors and integration of separation data with NMR, mass spectrometry, calorimetry, and molecular modeling to resolve the mechanisms of enantioselective recognition [76]. Thus, refs. [72,73,74,75] are retained for their historical and MTX-specific relevance, rather than as current benchmarks for analytical performance.

### 9.2. Sensors and Environmental Capture

More recent CD–MTX analytical applications have shifted toward surface-based sensing and analyte preconcentration rather than further development of MTX enantioseparation. These approaches address different analytical questions and should not be interpreted as direct successors to the chiral methods discussed above. Cyclodextrin–graphene nanosheets increased the electrochemical signals of MTX and doxorubicin and enabled trace analysis in a glassy carbon sensor [77]. β-CD-modified silver nanoparticles amplified the SERS signal of MTX through analyte preconcentration near the plasmonic surface [78]. A magnetite/graphene oxide adsorbent functionalized with β-CD removed MTX and doxorubicin from water [79]. These studies confirm that CD can increase local affinity for MTX, but they do not provide evidence about the safety or performance of a therapeutic dosage form.

### 9.3. Safety of Cyclodextrins and Potential Clinical Interactions

The safety profile of a cyclodextrin is derivative-, dose-, and route-dependent and should not be generalized across the class. Native β-CD has low aqueous solubility and is unsuitable for routine parenteral use because of renal toxicity, whereas HP-β-CD and SBE-β-CD have substantially greater clinical precedent as parenteral excipients [80,81,82]. Methylated β-CD derivatives can show greater membrane activity and cholesterol extraction and therefore require particular caution, especially for systemic administration. This distinction is relevant to the MTX literature because the derivative showing the largest oral pharmacokinetic improvement in the spray-dried comparison was DM-β-CD [25]; superior complexation or bioavailability does not by itself establish the most favorable safety profile. Likewise, amino-substituted, polymerized, crosslinked, grafted, and nanoparticulate CDs cannot automatically inherit the toxicological record of soluble HP-β-CD or SBE-β-CD. Their hemolysis, membrane effects, renal handling, impurities, degradation products, tissue persistence, and repeated-dose toxicity require formulation-specific assessment.

A 2022 case report described delayed MTX elimination after voriconazole was switched from the oral to the intravenous formulation, which contains sulfobutylether-β-CD (SBECD) [83]. The same report noted an earlier clinical observation of delayed high-dose MTX elimination during concomitant voriconazole treatment; however, the formulation and timing of voriconazole administration were not sufficiently documented to establish exposure to SBECD. In the 2022 study, direct experiments in rats showed only a marginal effect of SBECD on MTX disposition, whereas a significant reduction in clearance was demonstrated for phenolsulfonphthalein, which was used as a surrogate substrate for renal organic anion transport. Thus, the proposed inhibition of renal MTX elimination by SBECD remains mechanistically plausible but unconfirmed.

Literature search identified no subsequent case series, published pharmacovigilance analysis, or direct mechanistic study confirming this specific interaction. The evidence should therefore be regarded as a single SBECD-specific clinical observation supported by indirect experimental data rather than as an established drug–excipient interaction. It is relevant primarily as a hypothesis-generating safety consideration during high-dose MTX therapy and should not be interpreted as evidence that a therapeutically relevant SBECD–MTX inclusion complex forms in vivo.

## 10. Critical Synthesis and Perspectives

### 10.1. What the Current Evidence Establishes

The literature supports several conclusions with different levels of confidence. Within the available direct, within-study comparisons of native cyclodextrins, β-CD shows substantially stronger MTX association than α- or γ-CD, although this represents a condition-dependent qualitative trend rather than a universal quantitative ranking [15,23]. More recent comparative studies provide stronger evidence that host modification can alter affinity: Giri et al. found substantially greater apparent association for DM-β-CD than for native β-CD, HP-β-CD, or M-β-CD under a common experimental design, while carbohydrate substitution and secondary-face dimerization provide additional examples of structure-dependent tuning of MTX recognition [16,25,26]. Extensive substitution or polymerization, however, does not necessarily improve binding and may reduce cavity accessibility [23].

Complexation can improve dissolution, protect MTX from light, and increase oral or local exposure. The strongest evidence comes from studies that connect quantitative binding or phase solubility with orthogonal solid-state methods and then examine biopharmaceutical performance [14,25]. Even in those studies, the clinically relevant variable is not the largest possible constant. A useful host must bind strongly enough to solubilize and protect MTX, yet release it on the timescale of absorption or target-site action.

### 10.2. Recurrent Weaknesses in the Literature

The most persistent problem is nomenclature. The “MTX–CD complex” is often used for a carrier in which CD complexes another structural component, or for a material in which MTX could occupy several non-CD domains. The dendrimer NOE study and the polymerized β-CD/cholesterol hydrogel make this distinction particularly clear [27,56]. More careful terminology would improve mechanistic interpretation without diminishing the technological value of the carrier.

A second weakness is the comparison of conditional constants as though they were universal molecular properties. Reports often differ in pH, salt concentration, temperature, intrinsic solubility, concentration range, and fitting model. Stepwise and overall constants are mixed, and values in M^−1^ and M^−2^ are occasionally placed in one rank order. Future publications should report raw phase solubility plots, uncertainty estimates, the chosen equilibrium model, and evidence for aggregation or higher stoichiometry.

A third weakness is overreliance on nonspecific characterization. Disappearance of an MTX endotherm, lower XRD intensity, or a shifted FTIR band can support formation of a new solid but cannot alone prove cavity occupancy. Two-dimensional NMR, calorimetry, competition with a high-affinity guest, cavity blocking, and carefully designed carrier controls would make many claims substantially stronger. The most useful experiments are not necessarily the most elaborate; they are the ones that discriminate among plausible mechanisms.

### 10.3. Translational Priorities

Translational studies should measure solubility, free drug fraction, release, and permeability in the same experimental framework. This would prevent the common inference that a higher dissolved concentration automatically produces higher absorption. Physiologically relevant barriers, tissue retention measurements, and kinetic models that include complex dissociation are especially important for topical and intestinal systems [11,28].

Biological comparisons need to be mechanism-resolving. For a complex embedded in a carrier, useful groups include free MTX, the isolated complex, a carrier without CD, a carrier with CD but without MTX, a physical mixture, and the complete formulation. Stimulus-responsive studies additionally require matched controls with and without the stimulus for both the carrier and free drug. This design is more demanding than a free drug comparison, but it is the only way to determine whether an apparent gain comes from complexation, delivery, the material itself, or the applied physical trigger.

Most efficacy data remain limited to cell lines and short rodent experiments. In rheumatoid arthritis models, the available MTX–CD studies span semi-quantitative clinical arthritis scores, paw thickness measurements, radiographic assessment, and, in more recent systems, mechanistic readouts of inflammatory microenvironment and macrophage polarization. These approaches provide useful evidence of anti-inflammatory and structural effects, but clinically analogous long-term joint function remains largely untested. Future studies should therefore compare local CD formulations with clinically relevant oral or subcutaneous MTX, quantify tissue and plasma pharmacokinetics, and incorporate validated functional outcomes such as gait, weight-bearing, mobility, or joint use measurements together with blinded structural and histopathological assessment. In oncology, therapeutic index comparisons at equivalent levels of MTX exposure are more informative than higher cytotoxicity at unequal intracellular doses. Chronic toxicity, immunogenicity, renal handling of the CD, and long-term dermal safety are largely missing from the current evidence base.

Manufacturability deserves equal attention. Multilayer particles and dual-responsive materials can generate impressive in vitro release profiles, but clinical development depends on reproducible substitution, narrow size distributions, removal of residual reagents, sterilization, shelf stability, and scalable processes. The 2026 β-CD-grafted nanocellulose system is a promising research platform, yet its moderate polydispersity and in vitro-only validation illustrate how far an inventive carrier can remain from a dosage form ready for comparative preclinical development [68].

CNS delivery represents a conspicuous gap in the MTX–CD literature. Although MTX is used in the treatment of selected CNS malignancies, and its limited brain penetration has motivated several non-CD delivery strategies, we identified no MTX–CD system specifically developed to enhance blood–brain barrier penetration, brain pharmacokinetics, or intracranial tumor targeting. This absence is notable because cyclodextrin-based CNS delivery is being actively explored for other therapeutic agents. Future MTX–CD studies in this setting should therefore measure not only total solubility, but also the uncomplexed MTX fraction, complex dissociation kinetics, transendothelial permeability, brain-to-plasma exposure, regional brain distribution, and neurotoxicity. Such experiments would directly test whether cyclodextrin complexation can improve CNS delivery rather than merely increasing the amount of MTX dissolved in the systemic compartment [84,85].

## 11. Conclusions

MTX and cyclodextrins form a chemically and pharmaceutically productive pairing, but the observed benefits occur at different mechanistic levels and should not be treated as interchangeable evidence of cavity-mediated effects. In relatively simple, directly characterized MTX–CD systems, the effects that can be most confidently associated with molecular complexation are increased apparent solubility and dissolution, altered solid-state behavior, and, in selected systems, protection against photodegradation. Even in these cases, preparation-induced amorphization, ionization, or changes in hydration may contribute and should be separated experimentally from host–guest binding. Pharmacokinetic changes provide a higher level of functional evidence but are more difficult to attribute mechanistically. The increased C_max_ and AUC reported for the early β-CD complex are consistent with improved dissolution and absorption following complexation, although the age and analytical limitations of that study preclude assigning the entire effect uniquely to cavity inclusion. Likewise, the within-study comparison of spray-dried β-CD derivatives supports an important contribution of host chemistry, but spray drying and loss of crystallinity remain additional determinants of performance.

Once a preformed MTX–CD complex is incorporated into a secondary delivery platform, mechanistic attribution becomes substantially less specific. The improvement in oral bioavailability reported for the β-CD/SMEDDS system reflects the combined effects of complexation, lipid solubilization, dispersion into small droplets, and surfactant-mediated transport. The marked increase in exposure and prolonged half-life observed with the γ-CD metal–organic framework should similarly be regarded as a property of the porous framework as a whole rather than as evidence of a superior γ-CD cavity effect. The same principle applies to niosomes, hydrogels, photothermal gold nanoparticle assemblies, nanoemulsion gels, and stimulus-responsive carriers, in which retention, controlled release, targeting, membrane interactions, or external triggers can dominate the biological outcome. Unless an otherwise identical carrier lacking cyclodextrin, a blocked-cavity control, or another mechanism-resolving comparator is included, efficacy or pharmacokinetic improvement should therefore be attributed to the complete formulation rather than specifically to MTX–CD inclusion.

Importantly, none of the reviewed MTX–CD therapeutic systems has yet been supported by human pharmacokinetic or clinical efficacy data. Evidence that CD-based formulation reduces MTX toxicity is also insufficient: improved exposure and formulation performance should not be equated with an improved therapeutic index, and the only notable reduction in a toxicity-related endpoint was reported for a covalent prodrug rather than a reversible inclusion complex. Human translation will require comparisons with clinically used MTX formulations at matched exposure, direct characterization of complex or conjugate fate after administration, derivative-specific cyclodextrin safety assessment, and repeated-dose toxicology.

Accordingly, future studies should be designed not only to demonstrate that an MTX–CD formulation performs better, but also to establish which component of the formulation is responsible for that improvement. The next advances are likely to come from simpler and more discriminating studies rather than from architecture alone. Orthogonal evidence of binding, consistent reporting of conditional constants, direct measurement of free MTX and dissociation, and controls that isolate the contribution of CD will make formulation results easier to compare. Covalent MTX–CD conjugates should remain a separate prodrug category, with emphasis on hydrolysis and product identity. With those distinctions in place, cyclodextrin chemistry can be used more rationally: not to maximize binding at any cost, but to create an association that is stable during manufacture and storage, protective during delivery, and sufficiently reversible where MTX must become pharmacologically available.

## Figures and Tables

**Table 1:** Representative studies of direct MTX binding by cyclodextrins. Association parameters are grouped according to binding model and dimensionality. 1:1 and site-specific association parameters expressed in M^−1^.

Host/System	Solution-State Binding Model/Stoichiometry	Reported Association Parameter	Units	Principal Evidence	Interpretation and Source	Quantitative Equilibrium or Binding Measurement	Spatially Informative NMR	Solid-State Characterization	Evidence Domains
β-CD	1:1	K_1:1_ = 469.5	M^−1^	Phase solubility; XRD; DSC; IR; molecular modelling	Foundational 1:1 complex; K_1:1_ is a historical conditional value. Later studies provided stronger solution-state evidence for inclusion. The reported pharmacokinetic and antitumor findings should likewise be interpreted as historical proof-of-concept because they predate current analytical and reporting standards [14].	✓	—	✓	2/3
Alkylated β-CD derivatives	1:1	Up to ≈ 952 for 2,6-DM-β-CD	M^−1^	Phase solubility; ^1^H NMR; DSC	Substituent position was more important than methyl content alone; 2,6-dimethylation produced the strongest association in the series [15].	✓	—	✓	2/3
β-CD, HP-β-CD, M-β-CD, and DM-β-CD	1:1	433.6–3114.7	M^−1^	Phase solubility; FTIR; DSC; XRD	Within-study comparison of β-CD and its derivatives; DM-β-CD showed the highest apparent affinity at 3114.7 M^−1^, versus 552.3 M^−1^ for native β-CD, 574.5 M^−1^ for M-β-CD, and 433.6 M^−1^ for HP-β-CD [25].	✓	—	✓	2/3
Glucosyl-β-CD and maltosyl-β-CD	1:1	630–637	M^−1^	UV global fitting; NMR; thermal analysis; molecular modelling	Simple carbohydrate substitution increased affinity relative to native β-CD; addition of a second glucose unit provided no further measurable gain [26].	✓	—	✓	2/3
Secondary-face β-CD dimer 1	One dimer:two MTX; two equivalent binding sites	K_site_ = 1.9 × 10^3^	M^−1^ per site	ITC; Job plot; 1D NMR; ROESY	Approximately 3.5-fold higher site affinity than native β-CD; ROESY suggested relatively shallow binding at the wider rim [16].	✓	✓	—	2/3
Secondary-face β-CD dimer 2	One dimer:two MTX; two equivalent binding sites	K_site_ = 1.3 × 10^3^	M^−1^ per site	ITC; Job plot; 1D NMR; ROESY	Approximately 2.4-fold higher site affinity than native β-CD; similar shallow binding mode to dimer 1 [16].	✓	✓	—	2/3
β-CD/triethanolamine ternary system	1:1 MTX:β-CD equilibrium	K_1:1_ = 1179.8	M^−1^	UV; ROESY; FTIR; DSC/TG; XRD	Triethanolamine enhanced solubilization through ionization, electrostatic interactions and host–guest binding. The larger β-CD excess used to isolate the solid product should not be interpreted as solution stoichiometry [17].	✓	✓	✓	3/3
β-CD in ι-carrageenan hydrogel	1:1	K = 736 ± 42	M^−1^	Phase solubility; rheology; release; membrane transport	β-CD increased MTX solubilization and release but reduced membrane transport under some conditions [28].	✓	—	—	1/3
β-CD used for topical MTX nanogel	1:1 in phase solubility analysis	K_1:1_ = 118.3	M^−1^	Phase solubility; FTIR; DSC; XRD; TGA; ^1^H NMR	Moderate reversible association used as the molecular basis of a topical nanoemulsion gel formulation for rheumatoid arthritis [29].	✓	—	✓	2/3

Evidence-domain definitions: “Quantitative equilibrium/binding” denotes phase solubility analysis yielding an interpretable association parameter, ITC, spectroscopic equilibrium fitting, or another quantitative solution-binding measurement. “Spatial NMR” denotes 2D ROESY/NOESY or equivalent through-space evidence capable of localizing the guest relative to internal cyclodextrin protons; 1D chemical-shift changes alone were not counted in this category. “Solid-state characterization” denotes comparison of an isolated product with appropriate controls using DSC, PXRD/XRD, solid-state spectroscopy, or related techniques. Checkmarks indicate that the relevant evidence domain was represented, not that the method alone proves cavity inclusion.

**Table 2:** Representative studies of direct MTX binding by cyclodextrins. Association parameters are grouped according to binding model and dimensionality. Higher-order association parameters and systems for which no directly comparable K_1:1_ was reported.

Host/System	Binding Model/Stoichiometry	Reported Association Parameter	Units	Principal Evidence	Interpretation and Source
β-CD monomer in the monomer/dimer/polymer comparison	1:1	≈1.6–1.95 × 10^3^	M^−1^	Phase solubility; capillary electrophoresis; NMR	Used as the monomeric reference in a direct comparison of cyclodextrin architectures [23].
β-CD dimer in the monomer/dimer/polymer comparison	Overall 1:2 association	β_2_ ≈ 1.8 × 10^6^	M^−2^	Phase solubility; NMR	Cooperative higher-order association. This value cannot be numerically ranked against K_1:1_ or site-specific constants expressed in M^−1^ [23].
β-polycyclodextrin	Apparent binding per accessible cavity	Method-dependent; lower than monomeric β-CD	M^−1^	Phase solubility; capillary electrophoresis; NMR	Polymerization did not guarantee stronger accessible affinity because crosslinking generated heterogeneous and partly inaccessible binding environments [23].
β-CD nanostructure	1:1 assumed by the authors	Not reported	—	UV–Vis; XRD; DLS; DFT/QTAIM	Approximately nine-fold increase in apparent solubility and pH/temperature-dependent release; absence of an experimental association constant limits quantitative comparison [30].

Notes: Association parameters are reported using the binding models and units given in the original studies. K_1:1_ and site-specific constants expressed in M^−1^ are not directly comparable with overall association constants for 1:2 assemblies expressed in M^−2^. Absolute values should also be compared cautiously across studies because apparent association constants depend on pH, temperature, ionic strength, concentration range, analytical method, and fitting model. Unless explicitly stated otherwise, stoichiometries and binding models reported in this table refer to solution-state association determined or inferred from phase solubility analysis, calorimetry, Job plots, spectroscopic fitting, or related measurements. Molar ratios used during preparation or isolation of solid products are not reported as molecular binding stoichiometries. Solid-state characterization can support formation of a distinct product but does not by itself establish the stoichiometry of an inclusion complex in solution.

**Table 3:** Evidentiary value of analytical techniques across the four mechanistic categories of MTX–cyclodextrin systems.

Analytical Approach	Category A: Direct Reversible MTX–CD Complex	Category B: Formulation Based on a Preformed MTX–CD Complex	Category C: CD-Containing Carrier Without Demonstrated MTX Inclusion	Category D: Covalent MTX–CD Conjugate
Phase solubility analysis	Moderate–strong for association. Can establish concentration-dependent solubilization, suggest stoichiometry, and yield an apparent association constant when an appropriate model applies; does not by itself prove cavity occupancy.	Important before formulation. Supports existence and stoichiometry of the preformed complex before incorporation into the secondary dosage form. Measurements on the final multicomponent formulation are less mechanistically specific.	Limited unless performed on an isolated MTX–CD interaction. Increased apparent solubility in a carrier can reflect partitioning, adsorption, ionization, or polymer effects rather than cavity binding.	Generally not diagnostic of covalent attachment. May characterize residual non-covalent interactions but does not establish conjugation.
ITC/quantitative calorimetry	Strong for direct molecular association. Provides stoichiometry, affinity, enthalpy, and entropy, although interpretation depends on the binding model and competing equilibria. Does not independently localize MTX inside the cavity.	Strong when applied to the preformed MTX–CD pair. Can establish binding before formulation; measurements in the complete carrier may contain contributions from several interactions.	Potentially informative but often difficult to interpret. Whole-carrier heats may reflect MTX binding to CD, polymers, lipids, surfaces, or other domains. Appropriate carrier controls are essential.	Not a primary proof of conjugation. May characterize secondary non-covalent interactions after synthesis.
1D ^1^H NMR/chemical-shift analysis	Moderate/supportive. Guest and host chemical-shift changes indicate changes in molecular environment but do not uniquely establish cavity inclusion or orientation.	Useful for confirming that the preformed complex resembles the characterized Category A system before incorporation. Spectral interpretation may become difficult after addition of polymers, surfactants, or lipids.	Supportive but nonspecific. Chemical-shift changes can arise from several carrier domains; assignment to the CD cavity requires additional spatial evidence.	Useful for structural characterization of the conjugate, particularly when new covalent linkages or substitution patterns can be assigned, but should be combined with MS and related analyses.
2D ROESY/NOESY or equivalent through-space NMR	Strongest direct spatial evidence for cavity involvement. Cross-peaks between MTX protons and internal CD H3/H5 protons can localize the guest and constrain orientation, although dynamic exchange and spin diffusion must be considered.	Strong evidence that the molecular complex existed before formulation. Persistence of the same contacts in the final dosage form can support retention of the complex but is not always experimentally accessible.	Potentially decisive for demonstrating local MTX–CD cavity occupancy. However, observation of such a contact does not by itself convert a multifunctional carrier into Category A/B; the overall architecture may remain Category C.	Usually secondary. Can characterize additional host–guest interactions within a conjugate but is not evidence of the covalent MTX–CD bond itself.
Job plot/stoichiometric titration	Supportive–moderate. Can suggest dominant stoichiometry but should not be used alone, particularly for systems with multiple equilibria.	Useful during characterization of the precursor complex.	Low specificity in heterogeneous carriers containing multiple binding sites and components.	Generally not relevant to proof of covalent conjugation.
DSC	Supportive solid-state evidence. Disappearance or displacement of an MTX thermal event can indicate altered crystallinity or molecular dispersion but cannot by itself prove inclusion.	Useful for comparing the isolated preformed complex, physical mixture, and final formulation. Changes after formulation may also arise from the carrier matrix.	Low specificity for MTX cavity occupancy. Thermal changes can result from amorphization, adsorption, polymer mixing, or nanoscale dispersion.	Supportive for changes in thermal behavior, but not sufficient to establish covalent structure.
PXRD/XRD	Supportive solid-state evidence. Loss or alteration of MTX diffraction peaks can demonstrate formation of a new solid phase or amorphization, but not specifically cavity inclusion.	Useful for documenting the physical state of the preformed complex and changes following incorporation into the dosage form.	Low specificity. Reduced crystallinity in nanoparticles, hydrogels, or polymer matrices does not locate MTX within a CD cavity.	Supportive only. A changed diffraction pattern does not prove covalent conjugation.
FTIR/Raman spectroscopy	Supportive. Band shifts or broadening can indicate altered chemical environment or hydrogen bonding but are rarely unique to cavity inclusion.	Supportive for complex characterization and compatibility studies.	Low specificity in multicomponent carriers, where overlapping bands and multiple interactions are common.	Potentially useful for detecting formation or loss of characteristic functional groups, but structural assignment requires complementary techniques.
Drug loading content/entrapment efficiency	Not evidence of inclusion by itself. High drug incorporation may occur without cavity binding.	Formulation performance metric, not proof that the preformed complex remains intact after incorporation.	Formulation-level metric only. Quantifies carrier-associated/retained MTX but does not determine whether MTX occupies a CD cavity.	Generally not applicable as proof of conjugation.
Release, dissolution, or permeability studies	Functional consequences of association, but not structural proof. Release/permeation may additionally reveal the free drug/complex equilibrium.	Important formulation performance evidence, but effects can reflect both complexation and the secondary carrier.	Platform-level evidence. Cannot distinguish cavity binding from adsorption, diffusion, matrix partitioning, or carrier disassembly without mechanism-resolving controls.	Important for prodrug behavior, particularly when release requires cleavage of the covalent linkage.
High-resolution MS/MALDI-TOF/LC–MS	Usually not required for reversible inclusion unless used to support composition; gas-phase complexes should not be equated automatically with solution-state inclusion.	Secondary role.	Secondary role for carrier composition.	Strong evidence for conjugate composition and degree of substitution, particularly when combined with NMR and chromatographic characterization.
Covalent structure NMR, chromatographic purity, hydrolysis and product identification studies	Generally not central.	Generally not central.	Generally not central.	Core evidence for Category D. Should establish the presence and location of covalent attachment, conjugate purity, cleavage kinetics, and the chemical identity of released MTX-containing products.

Notes: The descriptors indicate the evidentiary value of a technique for the specific mechanistic question and should not be interpreted as a validated numerical quality score. No single method is sufficient to establish a classical inclusion complex. For Category A, the strongest evidence is obtained by combining quantitative solution-state association measurements with spatially informative NMR and, where an isolated solid is claimed, orthogonal solid-state characterization. For Category B, characterization should establish the MTX–CD complex before its incorporation into the secondary dosage form. For Category C, demonstration of local MTX–CD cavity contact does not necessarily reclassify the entire multifunctional carrier as Category A or B. For Category D, covalent linkage and cleavage/product identity take precedence over classical host–guest measurements.

**Table 4:** Selected secondary dosage forms built from a characterized or preformed MTX–CD complex.

Formulation	Role of the Complex	Principal Result	Model	Main Limitation and Source
HP-β-CD niosomes	Preformed MTX–HP-β-CD complex in aqueous niosome compartment	Entrapment 89% vs. 67% for non-complexed MTX; slower release; improved stability and life-span response	Tumor-bearing BALB/c mice; 5 mg kg^−1^	Historical study from the same institutional research program as ref. [37]; no post-2015 independent MTX–CD niosome replication identified; limited analytical and batch-level reporting [36]
β-CD niosomes	Preformed MTX–β-CD complex followed by lipid-layer hydration	Entrapment 84% vs. 67% for non-complexed MTX; slower release; improved stability and tumor response	C57BL/6J mice bearing B16F1 melanoma; 10 mg kg^−1^	Distinct in vivo experiment but closely related formulation methodology and research provenance to ref. [36]; no post-2015 independent MTX–CD niosome replication identified [37]
β-CD complex in SMEDDS	Freeze-dried complex dispersed in a self-emulsifying lipid phase	About ten-fold solubility gain; 1.57-fold bioavailability; photoprotection	Rat oral PK	Preparation ratio should not be read as molecular stoichiometry [32]
β-CD/MTX-gold nanoparticles	Preformed complex coupled to photothermal gold particles	Light-triggered release and recovery of cytotoxicity	HeLa cells	Partial release; no in vivo assessment [18]
γ-CD-MOF	MTX loaded into a porous γ-CD framework	Higher Cmax and markedly increased AUC; longer half-life	Rat PK and in vitro testing	Framework effects cannot be reduced to cavity inclusion [34]
β-CD-crosslinked gelatin	CD serves as crosslinker and reversible host	Higher loading; burst release reduced to 10–30%	In vitro release	No biological efficacy data [35]
Iota-carrageenan/β-CD	Complex formed within a topical hydrogel	Approximately ten-fold solubility gain; rapid release	Membrane transport model	Transport was lower when complexation was strong [28]
Kappa-carrageenan/β-CD	Variable CD content in hydrogel	Up to complete release within 4 h	In vitro release and membrane transport	Increasing CD also reduced permeation [33]
Modified-CD/iota-carrageenan	Comparison of β-, HP-, and amino-β-CD	Amino-β-CD most strongly changed solubility and gel structure	In vitro	Binding and network effects are inseparable [31]
β-CD/MTX nanoemulsion gel	Preformed complex in nanoemulsion and Carbopol matrix	Release 78%; permeation 84%; improved arthritis outcomes	Porcine skin and CFA rat model	Small groups and no long-term safety/PK [29]

Note: References [36,37] originated from the same institutional research program and used closely related niosomal methodology. Although they employed different cyclodextrin hosts and distinct in vivo models, they should be regarded as related rather than fully independent evidence. No post-2015 MTX–CD niosome study independently reproducing the reported enhancement in entrapment efficiency was identified. All systems summarized in this table remain preclinical; no human pharmacokinetic study or clinical trial of an MTX–CD therapeutic formulation was identified. Reported efficacy or pharmacokinetic improvement should not be interpreted as evidence of reduced toxicity unless toxicity was evaluated independently.

**Table 5:** Functional roles of CD in selected MTX carriers and covalent constructs.

System	Class	Role of MTX and CD	Interpretive Limitation and Source	Reclassification Potential
Porphyrin–CD conjugate	Multimodal carrier	Drug binding plus photodynamic therapy	Outcome depends on the whole optical/therapeutic platform [38]	Remain C: direct MTX–CD binding could occur, but the complete system is intrinsically multimodal
β-CD/PAMAM star polymer	Drug/siRNA co-delivery	CD core and cationic dendrons provide different binding domains	MTX localization requires direct NMR evidence [40]	Potential A: direct spatial evidence localizing MTX to the central β-CD cavity could establish a discrete host–guest interaction
PLGA-β-CD nanoparticles	Polymeric nanoparticles	MTX encapsulation and modified release	Effect of β-CD is not isolated from PLGA particle effects [44]	Remain C: cavity binding could be demonstrated, but β-CD is integrated into a polymeric nanoparticle
GO-polymer brush-β-CD	pH/temperature-responsive carrier	Pendant CD and polymer brush retain MTX	Guest location is inferred rather than directly mapped [45]	Remain C: possible cavity interaction represents only one binding mode within the composite carrier
Magnetic CD nanoparticles	Magnetic/dual drug carrier	MTX alone or with tamoxifen	Magnetic targeting and carrier chemistry confound cavity effects [47,48]	Remain C: magnetic core, surface chemistry, and other binding domains remain integral to drug delivery
Poly(α-CD)/β-CD-azobenzene	pH/UV-responsive nanoparticles	MTX is a physically loaded model payload; DLC 2.09% and EE 21.3% describe whole-nanoparticle drug retention	The demonstrated host–guest pair is poly(α-CD)/azobenzene; DLC and EE do not establish MTX occupancy of a CD cavity [59]	Remain C: the demonstrated host–guest interaction is part of carrier assembly and involves azobenzene rather than MTX
Polymerized β-CD/PEG-cholesterol gel	Injectable host–guest hydrogel	MTX and 5-FU are dispersed in the network	The demonstrated guest is cholesterol, not MTX [56]	Remain C: cholesterol, rather than MTX, is the demonstrated CD guest responsible for network formation
β-CD nanosponges	Site-retentive topical nanogel	MTX retained in a crosslinked polycyclodextrin network	Nanosponge and gel contributions are difficult to separate [58]	Remain C: MTX may interact with cavities, but retention occurs within a crosslinked heterogeneous network
Am7CD/SDS nanoparticles	Oral pH/temperature-responsive carrier	MTX is protected and released in the intestinal environment	Several possible binding domains; no in vivo study [60]	Potential B: could be reclassified if a discrete MTX–Am7CD complex is demonstrated before incorporation into the SDS-containing carrier
POM/β-CD assembly	Ultrasound-responsive RA system	MTX delivery combined with ROS scavenging	POM, CD, ultrasound, and MTX act jointly [61]	Remain C: therapeutic behavior depends on the multicomponent POM/CD/ultrasound platform
β-CD-grafted nanocellulose	pH/temperature-responsive co-delivery	MTX and curcumin loaded by multiple interactions	In vitro only; MTX position unresolved [68]	Remain C: direct MTX cavity occupancy could be demonstrated, but β-CD remains integrated into a multifunctional polymeric carrier
MTX-α/γ-CD esters	Covalent colon-directed prodrugs	CD masks carboxyl groups until hydrolysis	Not inclusion complexes [69]	Remain D: MTX is covalently linked to the CD scaffold; the system is a prodrug rather than a reversible inclusion complex
MTX-α/β/γ-CD conjugates	Enzymatic covalent conjugates	Ultrasound-assisted multivalent esterification	No release, stability, or efficacy study [70]	Remain D: covalent MTX–CD attachment defines the mechanistic category irrespective of possible additional non-covalent interactions.
MTX-β-CD/biotin-adamantane construct	Covalent conjugate plus secondary host-guest assembly	MTX is linked to CD; adamantane is the guest	Requires separate nomenclature for both interactions [71]	Remain D at the MTX–CD level: MTX is covalently attached to CD, whereas the separately demonstrated host–guest interaction involves β-CD and adamantane

Note: “Potential A/B” denotes the possibility of reclassification of the complete system after additional mechanistic evidence. “Remain C” does not exclude the presence of an MTX–CD host–guest interaction; rather, it indicates that such an interaction would represent only one component of a larger carrier architecture. Drug-loading content and entrapment efficiency reported for Category C systems are formulation-level metrics unless the primary study independently demonstrates localization of MTX within a cyclodextrin cavity. These parameters should not be interpreted as evidence of inclusion by themselves. All representative Category C systems remain preclinical, and no human MTX pharmacokinetic or clinical comparison was identified.

## Data Availability

The original contributions presented in this study are included in the article. Further inquiries can be directed to the corresponding author.
